# Polyphasic *in vitro* characterization of the pigment-producing microfungus *Rhodotorula* sp. for potential application as a probiotic in mariculture

**DOI:** 10.3389/fnut.2026.1759183

**Published:** 2026-05-07

**Authors:** Qurat ul Ain, Pooja Baiju, Saima Rehman, Kajal Chakraborty, Ashwin A. Pai, S. Chandrasekar, Sanal Ebeneezar, D. Linga Prabu, T. D. Unnimaya, S. Shylaja, Adnan H. Gora

**Affiliations:** 1 Marine Biotechnology, Fish Nutrition and Health Division, ICAR-Central Marine Fisheries Research Institute, Kochi, India; 2 Department of Biotechnology, Cochin University of Science and Technology, Kochi, India

**Keywords:** characterisation framework, probiotics, carotenoids, EPA, fatty acids, microfungi, *Rhodotorula* sp.

## Abstract

Pigment-producing microfungi represent a promising source of bioactive compounds. This study represents a polyphasic evaluation of a novel *Rhodotorula paludigena* (PX945103) isolate to determine its suitability as a probiotic with nutritional, functional, and biotechnological potential. The pigment producing microbe was isolated from the mangrove ecosystems of Kochi, southern India. The isolate was identified as *Rhodotorula* sp. based on morphological and molecular characteristics. Microbial characterization of the isolate revealed stable growth performance, with the strain producing a dry biomass of about 0.73 g/L in YEPD medium. It also synthesized pigments up to 398.7 μg/g dry weight and 1681.56 μg/L culture. The extracted pigment exhibited a λ_max_ at 458 nm, with maximum intracellular carotenoid accumulation occurring on 5th day of cultivation. Fatty acid profiling of the *Rhodotorula paludigena* (PX945103) indicated PUFA content of about 28.21% of the total fatty acids. Linoleic acid and *α*-Linolenic acid dominated the PUFA profile accounting for 19.9 and 4.1% of the total fatty acids, respectively. Amino acid profiling of the *Rhodotorula* sp. revealed a lysine content of 61.86 g/kg dry weight. Incubation in simulated intestinal fluid had no significant impact on viability of the microbe. However, in simulated gastric fluid the viability of *Rhodotorula* sp. decreased significantly after 80 min, from 11.76 to 9.52 ± 0.14 Log₁₀ CFU/mL. A strong correlation was observed between the biomass and radical scavenging activity of the isolate (R^2^ = 0.98, *p* < 0.0001 for DPPH; R^2^ = 0.91, *p* < 0.0001 for ABTS). Well diffusion assay of the crude acetone extract of *Rhodotorula paludigena* (PX945103) was effective against pathogens, with zones of inhibition of 17.66 ± 0.44, 15.00 ± 0.57, 25.30 ± 0.33 and 15.00 ± 0.57 mm for *V. parahaemolyticus, V. alginolyticus, V. harveyi and E. coli*, respectively indicating considerable antibacterial effects. This study establishes a foundational framework for advancing *Rhodotorula* sp. as a promising next-generation probiotic microfungus.

## 1 Introduction

Over the last three decades, the global aquatic animal farming sector has expanded at an average growth rate of 6.7% (1, 2). A major milestone was achieved in 2022 when aquaculture production surpassed capture fisheries in aquatic animal production, producing 94.4 million tonnes amounting to 51% of the aquatic animal production (2). In the future, this sector will continue to represent a crucial means of satisfying the increasing food demand and addressing nutritional deficiencies and minimizing environmental impact compared to many animal-based food production systems (3). While production has increased substantially, the aquaculture industry has predominantly shifted towards more intensive culture systems that have brought unique challenges, connected to animal welfare and disease resistance. Antibiotics were the standard and widely accepted bacterial control agents in aquaculture for almost three decades until evidence about their risks to the consumers and the environment was presented (4, 5). To circumvent the challenge of antimicrobial resistance, a tremendous amount of research focus has been on alternative disease resistance strategies including the use of probiotics (6, 7).

Probiotics have demonstrated promise as sustainable tools that can bolster antioxidant capacity, disease resistance, and nutrient assimilation, in farmed aquatic animals (8, 9). These beneficial microbes can also be used to improve growth performance, enhance feed conversion and immune function, and improve water quality in aquaculture (10). Such functional traits are increasingly considered the defining features of effective aquaculture probiotics (11, 12). Historically, probiotic research and applications have focused predominantly on bacterial species, particularly lactic acid bacteria (e.g., *Bacillus* and *Lactobacillus* spp.) (13, 14) and fungi such as *Saccharomyces cerevisae* and *Saccharomyces boulardii* (15, 16). The effects of these probiotics is well-documented across diverse host species. However, in recent years there has been a growing interest in novel probiotics, as alternative or complementary functional additives in aquafeeds. Among these, *Rhodotorula* spp. have attracted increasing attention because of their capacity to produce carotenoids, and other antioxidant metabolites (17). Notably, dietary supplementation with *Rhodotorula* spp. has been reported to enhance growth performance, feed utilization, immune parameters, and oxidative stress resistance in different fish species (18, 19) highlighting their promising potential as a functional fungal probiotic in aquaculture systems.

**Figure 1:**
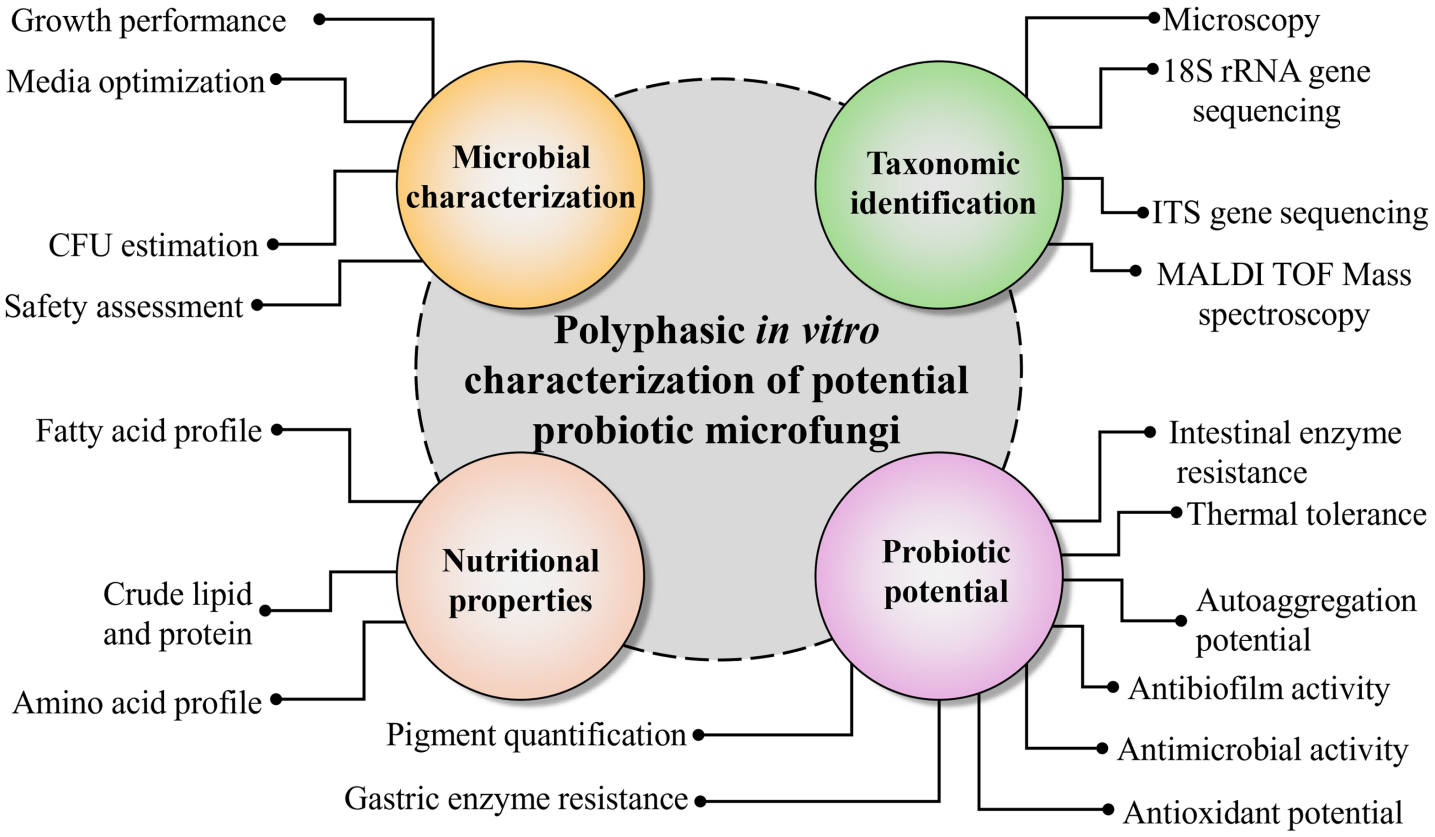
Overview of the polyphasic characterization framework applied to potential probiotic microfungi. The workflow begins with isolation and preliminary screening, followed by four major characterization components: (1) microbial characterization, including safety assessment, growth performance, media optimization, and CFU estimation; (2) taxonomic identification, involving microscopy, 18S rRNA and ITS sequencing, and MALDI-TOF mass spectrometry; (3) nutritional properties comprising fatty acid profile, amino acid composition, and proximate analyses; and (4) probiotic potential, assessed through gastric and intestinal resistance, antimicrobial and antibiofilm activity, autoaggregation capacity, and antioxidant potential. Together, these components provide a comprehensive polyphasic evaluation of candidate probiotic microfungi.

While many probiotic strains are designed for specific purposes, only a few provide broad benefits that include immune support, antioxidant activity, and nutritional improvement (7, 8). This necessitates using a combination of different probiotic strains and prebiotics which can produce stronger and more targeted effects than using single probiotics alone. Though preliminary guidelines are available (20) most studies focus on isolated characteristics such as antimicrobial activity or gut survivability, without integrating nutritional, taxonomic, and probiotic traits into a single, coherent assessment. There is currently no well-developed standardized framework for systematically evaluating potential probiotic strains before *in vivo* application. This gap limits the comparability of results across studies and slows progress in identifying broad-spectrum and effective probiotic candidates for aquaculture systems. To address this gap, we developed an *in vitro* evaluation framework for assessing potential probiotics for aquaculture. This framework integrates five core principles: microbial characterization, *in vitro* pathogenicity assessment, multilayered taxonomic identification, nutritional profiling, and typical probiotic traits (Figure 1). We applied this framework to a novel *Rhodotorula* strain from mangrove leaves to evaluate its suitability as a potential probiotic.

## 2 Materials and methods

### 2.1 Preliminary screening and isolation

Senescent mangrove leaves (SML) were collected from the Krishi Vigyan Kendra of ICAR-Central Marine Fisheries Research Institute, Njarakkal, Kochi (10°02′38″N 76°12′46″E) (Supplementary Figure 1A). Dead and senescent leaves in different stages of decay were transported to the laboratory in a sterile zip-lock bag and kept at 4 °C until further processing. The SML were cleaned with sterile seawater and then used for isolation of yeast species by direct plating method (21). The SML were then cut aseptically into 0.5 cm^2^ fragments and transferred to a YEPD agar Petri dishes (yeast extract: 10 g L^−1^, peptone: 20 g L^−1^, and glucose: 20 g L^−1^) in NSW along with antibacterial agents (ampicillin 100 μg mL^−1^, streptomycin sulfate 50 μg mL^−1^, and kanamycin sulphate 30 μg mL^−1^) to avoid bacterial contamination. The plates were incubated at 28 °C, pH 7.2, for 4 days and checked daily for microbial growth. After 2 to 3 days of incubation, creamy orange colonies began appearing beneath the leaf cutouts on the YEPD agar plates. These colonies were aseptically transferred to fresh plates to obtain pure cultures. The pure colonies of the mother culture were cryopreserved at −80 °C in 20% glycerol stock for long-term storage. To exclude the possibility of immediate pathogenic effects, the isolate was additionally cultured on sheep blood agar (Himedia, Thane, India; catalogue number: MP1301) for hemolysis evaluation. Since pathogenic microbes produce extracellular tissue degrading enzymes (22, 23) we also evaluated the ability of the isolated *R. paludigena* for extracellular protease, lipase and phospholipase activity. Extracellular protease activity was ruled out by spot plating on skimmed milk agar (Himedia, Catalogue number: M763). Similarly, phospholipase activity was evaluated by spot plating a loopful of *R. paludigena* on egg yolk agar base (Himedia, Catalogue number M808) containing 10% w/w egg yolk. Lipase activity was evaluated using agar plates prepared as follows: peptone (8 gL^−1^), calcium chloride (1 gL^−1^), agar (15 gL^−1^), and Tween 80®(4 mL/L^−1^) (24). For all extracellular enzyme screening tests, the plates were incubated at 28 °C, for 4 days and checked daily for microbial growth and appearance of media clearance zones that indicate positive reactions.

### 2.2 Microbial characterization

The growth curve analysis of *Rhodotorula* was performed to determine its growth pattern and identify the early stationary phase for downstream applications (25). *Rhodotorula* was inoculated into 150 mL of sterile YEPD broth and incubated at 28 °C. At 4 to 8-h intervals, 1 mL aliquots were withdrawn, centrifuged at 5000 × g for 10 min at room temperature, and washed twice with sterile phosphate buffered saline (PBS) of pH 7.4. The final cell pellet was resuspended in 1 mL of PBS, and 200 μL of the suspension was transferred to a 96-well microtiter plate. Optical density (OD) was measured at 600 nm using PBS as the blank. The growth curve was further analyzed to correlate OD with viable cell counts. For this, cultures grown in YEPD broth were subjected to serial dilution and spread plate analysis. Plates were incubated at 28 °C for 24 h, and colony-forming units per mL (CFU/mL) were calculated. Pure cultures were also inoculated into three different media YEPD, SDA (peptone: 10 g L^−1^, glucose: 40 g L^−1^), and LB (tryptone: 10 g L^−1^, yeast extract: 5 g L^−1^, NaCl: 10 g L^−1^) broths and incubated for five days. After cultivation, the biomass was harvested by centrifugation at 5000 × g for 10 min and lyophilized. The dry biomass was measured using an analytical balance (Nakagyo-ku, Kyoto, Japan).

### 2.3 Taxonomic identification

#### 2.3.1 Microscopy

To understand the morphology of the cultured yeast, brightfield and electron microscopy were performed. For brightfield microscopy, a loopful of pure culture was taken and placed on a glass slide. The culture was diluted with 20–50 μL of PBS and covered with a glass coverslip. Imaging was performed using a Leica DM2500 microscope (Leica Microsystems, Inc., Wetzlar, Germany) at magnifications of 400 × and 1,000×. For electron microscopy pure culture was obtained by culturing the yeast in YEPD broth and harvesting the culture in early-stationary phase by centrifugation. Scanning electron microscopy (SEM) technique was employed using the freeze-dried yeast cells (25). Briefly, the yeast cells were placed onto a glass slide, fixed with 2.5% v/v glutaraldehyde at 4 °C for 2 h, and then washed thrice with 0.1 M sucrose in 0.1 M cacodylate buffer, pH 7.2 for 4.5 min. The sample was post-fixed with 2% OsO_4_ at retention time for 1 h 30 min under the flow-hood, followed by three washes with distilled water lasting for five minutes each. After fixation, the samples were dehydrated with varying grades of ethanol (10, 20, 40, 60, 80, 90, and 100%), and then isoamyl acetate was used for substitution. CO_2_ was used to dry the mounted samples after mounting on carbon-taped aluminum stubs. Following the application of a sputter-coated with gold, the samples were observed under a SEM at 3000 × magnification.

#### 2.3.2 Phylogenetic analysis of 18S rRNA and ITS gene

The molecular identification of isolated isolate was performed using the method described by Mo et al. (26), with some changes. Briefly, 5 mL of early stationary phase culture was harvested by centrifuged at 5,000 × g at 4 °C for 10 min. The DNA was then extracted in chloroform-isoamyl alcohol and precipitated by chilled isopropanol (27). The purified DNA was dissolved in 30 μL of TE buffer and stored at −20 °C until use. The 18S rRNA gene was amplified using universal primers as follows: 18S Univ F -5′-TGGTTGATCCTGCCAG-3′ and 18S Univ R -5′ TAATGATCCTTCCGCAGGTTCACCT-3′. Each polymerase chain reaction (PCR) mixture (25 μL) protocol consisted of 1 μL of template DNA, 10.5 μL of Nuclease free water, 12.5 μL of PCR Mastermix, and 0.5 μL each of Forward and Reverse Primer. The PCR program included initial denaturation at (94 °C for 5 min) followed by 35 cycles of denaturation (94 °C for 30 s), annealing (45 °C for 30 s) and extension (72 °C for 1 min). Final extension was carried out at (72 °C for 7 min). The amplicons along with a 100 bp ladder were examined by electrophoresis on 1.2% agarose gel and were made visible by ethidium bromide staining. The *internal transcribed spacer* (ITS) gene was amplified using universal primers as follows: F -5′-TGGTTGATCCTGCCAG-3′ and R -5′ TAATGATCCTTCCGCAGGTTCACCT-3′ (28). Each polymerase chain reaction (PCR) mixture (25 μL) protocol consisted of 1 μL of template DNA, 10.5 μL of Nuclease free water, 12.5 μL of PCR Mastermix, and 0.5 μL each of Forward and Reverse Primer. The PCR program included initial denaturation at (94 °C for 5 min) followed by 35 cycles of denaturation (94 °C for 30 s), annealing (55 °C for 30 s) and extension (72 °C for 1 min). Final extension was carried out at (72 °C for 7 min). The sequences of the DNA products were determined by GeneSpec (Kochi, India). The sequencing was performed on a using a Roche 454 pyro sequencer (Basel, Switzerland). Following the alignment, a phylogenetic tree was constructed based on the aligned sequences. Phylogenetic analysis was carried out using MEGA version 11. The phylogenetic tree was constructed by the neighbor-joining (NJ) method with 1,000 repetitions of bootstraps (29).

#### 2.3.3 MALDI TOF mass spectroscopy

Well-isolated pure colonies of the yeast were obtained from routine YEPD agar plates. Sample preparation for matrix-assisted laser desorption/ionization time-of-flight mass spectrometry (MALDI-TOF MS) was performed using the ethanol/formic acid extraction method as described in the manufacturer’s protocol (Autof MS1000 system, Zhengzhou, China) and following the Clinical and Laboratory Standards Institute (CLSI) guideline M58 (30). Briefly, one single colony from a fresh culture was transferred into a 1.5 mL microcentrifuge tube containing 300 μL of deionized water and vortexed to obtain a uniform suspension. Subsequently, 900 μL of 100% HPLC-grade ethanol was added, mixed thoroughly, and centrifuged at 10000 × g for 2–5 min at room temperature. The supernatant was discarded, and the centrifugation step was repeated to remove residual ethanol completely. The resulting pellet was dried at 37 °C for 5 min until completely dry. The dried pellet was resuspended in 10 μL of lysis reagent 1 (MSA01, Autof MS Reagent Kit) and incubated at room temperature for 30 min, followed by the addition of 10 μL of lysis reagent 2 (MSA02, Autof MS Reagent Kit). After vortexing and incubation for another 30 min, the suspension was centrifuged again for 5 min at room temperature (8,000 × g). A 1 μL aliquot of the supernatant was spotted onto a clean stainless-steel target plate and air-dried. Subsequently, 1 μL of the matrix solution (MSA03, Autof MS Reagent Kit) was added to each spot and allowed to dry at room temperature. The prepared target plate was loaded into the Autof MS1000 instrument for spectral acquisition and microbial identification within 2 h of preparation. Results were interpreted based on the log score value of the first best match following manufacturer’s instructions as follows: 0.0 ≤ log score < 6.0: not reliable identification; 6.0 ≤ log score < 9.0: genus-level reliable identification and probable species-level identification; log score ≥ 9.0: species-level reliable identification (31).

### 2.4 Typical probiotic potential

#### 2.4.1 Antioxidant potential

Antioxidant potential of the whole freeze-dried biomass and crude acetone extract of *Rhodotorula* sp. was evaluated through stable free radical 2,2-diphenyl-1-picrylhydrazyl (DPPH) (Sigma-Aldrich) assay and 2,2′-azino-bis(3-ethylbenzothiazoline-6-sulfonic acid) (ABTS) assay kit (Sigma-Aldrich) using Ascorbic acid as a standard, following previously described protocols (32). Briefly, 2 mL of 0.06 M methanolic DPPH was added to crude acetone extract, or whole lyophilized biomass, of *Rhodotorula*, mixed well and kept in the dark for 30 min. Then the absorbance was measured at 517 nm against the reagent blank as control. The ability of each concentration of the crude acetone extract or the whole *Rhodotorula* sp. biomass to scavenge DPPH radical was expressed as percentage (%) inhibition.

#### 2.4.2 Simulated gastric and intestinal juice challenge

Simulated gastric fluid (SGF) was prepared by dissolving 2 g of sodium chloride (NaCl) in approximately 800 mL of deionized water, followed by the addition of 2 mL of concentrated hydrochloric acid (25). The volume was then made up to 1 L with deionized water and mixed thoroughly to obtain the SGF stock solution. For enzymatic simulation, 0.064 g of pepsin was dissolved in 20 mL of the SGF stock, and the pH of the resulting solution was adjusted to 2.5 using dilute HCl or sodium hydroxide (NaOH) as necessary. Simulated intestinal fluid (SIF) was prepared by dissolving 10 g of trypsin, 10 g of pancreatin, 3 g of bile salts, and 8.5 g of sodium chloride in 1 L of deionized water. Freshly harvested *Rhodotorula* culture (5.4–11.7 × 10^11^ CFU/mL) was suspended in 200 μL of the freshly prepared SGF and SIF. The samples were incubated at 29 °C with agitation at 100 rpm. Aliquots were collected at 0, 40, and 80 min. At each point, samples were subjected to serial dilution in sterile PBS. From each dilution, 100 μL was plated onto sterile YEPD agar plates. Plates were incubated at 29 °C for 24 h. After incubation, CFU were counted to determine the number of viable bacteria at each time interval.

#### 2.4.3 Antimicrobial activity

Antibacterial activity was evaluated using the agar disc diffusion method as previously described (33). Four pathogens, *Vibrio harveyi*, *V. parahaemolyticus*, *V alginolyticus,* and *E. coli* were taken as the test cultures. Additionally, a mixed culture treatment containing all four pathogens was also included. The pathogens were sub-cultured twice to revive the culture from the stock. Chloramphenicol discs (30 μg) were used as a positive control, and acetone served as a solvent control to compare the suppression of pathogenic growth. The pathogenic bacterial lawn was cultivated on YEPD agar plates. Subsequently, the crude acetone extract of *Rhodotorula* sp. were applied onto sterile discs and placed on plates. The plates were air-dried under a laminar airflow hood and then incubated at 29 °C. The antibacterial activities were evaluated by measuring the zone of inhibition (mm) using an antibiotic zone scale after 24 h of incubation.

#### 2.4.4 Antibiofilm activity

Antibiofilm activity was assessed using the standard crystal violet microtiter plate assay as previously described (34, 35). To determine antibiofilm activity of *Rhodotorula* sp., four different concentrations of culture between 1.25 × 10^5^ to 1 × 10^6^ CFU/mL were used against four different pathogenic bacteria, *Vibrio harveyi* (MTCC 7954) [(1.45–1.82) × 10^5^ CFU/mL], *V. parahaemolyticus* (MTCC 451) [(5.28–5.52) × 10^8^ CFU/mL], *V alginolyticus* (MTCC 4439) [(1.28–1.93) × 10^8^ CFU/mL] and *E. coli* (MTCC 40) [(2.53–2.58) × 10^8^ CFU/mL] and a mixed pathogen group (OD_600_: 0.319 ± 0.003) in different ratios (Supplementary Table 1). Briefly, 100 μL of pathogenic culture was mixed with different concentrations of *Rhodotorula* in a 96 well-plate. The culture was incubated at 29 °C overnight. The next day, the culture was discarded, and biofilm formation in the wells was confirmed by crystal violet staining. Briefly, the wells were flooded with 0.1% (v/v) crystal violet solution, followed by washing the wells twice with PBS. Finally, the crystal violet bound to the developed biofilm was solubilized using 70% ethanol, and the optical density (OD) of the wells was measured using a 96-well plate reader spectrophotometer (Thermo Fisher Multiskan SkyHigh). The percentage of antibiofilm activity was calculated using the following formula:


$$\text{Antibiofilm activity}\;(\%)=(A_{\text{control}}–A_{\text{treated}})\times 100/A_{\text{control}}$$

where *A_control_* = absorbance (600 nm) of the untreated biofilm, and *A_treated_* = absorbance (600 nm) of the biofilm treated with *Rhodotorula*.

#### 2.4.5 Autoaggregation

Freshly grown *Rhodotorula* cells were harvested by centrifugation at 5000 × g for 10 min, washed, and resuspended in PBS (pH 7.2). The cell suspension was adjusted to OD_600_ of 1.0. The yeast cells were then incubated at 29 °C without stirring for 30 min for up to 4 h, and at 24 h. Following incubation supernatant fraction of the culture was transferred to a fresh 96-well plate and subjected to centrifugation at 5000 × g for 10 min. The settled cells were resuspended in 100 uL of PBS and the absorbance (A_t_) was recorded using a microplate spectrophotometer (Agilent Technologies, Santa Clara, CA, United States). The autoaggregation ability was calculated using the following formula (36, 37):


$$\text{Auto}-\text{aggregation}\%=[1-(A_{t}/A_{0})]\times 100$$

where *A_t_* is the absorbance (600 nm) at each interval time, and *A_0_* is the absorbance at the initial time.

#### 2.4.6 Pigment estimation

For pigment extraction, fresh cultures of *Rhodotorula* were harvested by centrifugation at 5000 × g for 5 min. Pigments were extracted using three solvents: acetone, n-hexane and dichloromethane. The cells were suspended in the solvents at a 1:10 (w/v) ratio and kept on a rocker overnight in the dark. The absorption maximum (λmax) of pigments in the different solvents was analyzed using a spectrophotometer (Thermo Scientific Multiskan SkyHigh, 11,550). Carotenoid content in dried cell pellets (μg/g dry weight) was calculated using the following equation (38, 39):

Mass fraction of carotenoid content (μg per g dry weight) = $\frac{A\times V(\mathrm{mL})\times 10^{6}}{A_{1\;\mathrm{cm}}^{1\%}\times 100\times m(g)}$
A = absorbance at 458 nm.
V = total extract volume.
m = dry cell biomass.
$A_{1\;\mathrm{cm}}^{1\%}$ = 2,500 (*β-carotene* extinction coefficient in acetone).

The volumetric carotenoid concentration (μg/L) was obtained by multiplying the mass fraction of total carotenoids (μg/g) by the biomass concentration (g/L).

### 2.5 Nutritional profiling

#### 2.5.1 Crude protein and crude lipid and ash estimation

The moisture content of the sample was calculated using the formula [(wet weight − dry weight) × 100]/wet weight. Crude protein content (N × 6.25) was conducted using the Kjeldahl system (FOSS Kjeltec, 2,300). Crude lipid content was determined by ether extraction using a Soxhlet system (FOSS Soxtec, 2043). Ash content was determined by incinerating the samples in a muffle furnace at 550 °C for 5 h.

#### 2.5.2 Amino acid analysis

Amino acid analysis was performed using the Pico-Tag method (40) on a Waters HPLC system (Waters Corp., Milford, MA, United States). *Rhodotorula* cultures grown in YEPD medium were harvested at the early stationary phase by centrifugation at 5,000 × g for 10 min and washed twice with PBS (pH 7.2). The cell pellets were then hydrolyzed with 10 mL of 6 N HCl at 110 °C for 24 h in sealed tubes. The hydrolysates were filtered (Whatman No. 1) and concentrated under reduced pressure at 100 °C using a rotary evaporator. The dried residue was reconstituted in 5 mL of 0.05 N HCl and filtered through a 0.2 μm nylon syringe filter (Whatman). Samples were redried with a methanol:water:triethylamine mixture (95:2:1, v/v/v) and then derivatized with freshly prepared phenylisothiocyanate (PITC; methanol:phenylisothiocyanate:triethylamine, 7:2:1, v/v/v) to obtain phenylthiocarbamyl (PTC) amino acid derivatives. The PTC derivatives (20 μL) were diluted with 200 μL of sample diluent (5 mM sodium phosphate buffer, pH 7.4:acetonitrile, 95:5, v/v) prior to HPLC injection. Chromatographic separation was achieved on a Nova-Pak C18 column (3.9 × 150 mm; Waters) maintained at 38 ± 1 °C. Detection was performed at 254 nm using a dual absorbance detector (Waters 2,487). The mobile phases consisted of Eluent A (0.14 M sodium acetate trihydrate (pH 6.4) containing 0.05% triethylamine and 6% acetonitrile) and Eluent B (acetonitrile:water, 60:40, v/v). A binary gradient program with increasing proportion of eluent B was applied, followed by a 100% Eluent B wash step and equilibration. Standard amino acid mixtures (Pierce Amino Acid Standard H; Thermo Fisher Scientific) were analyzed before each batch. Quantification was performed by comparing peak areas of samples with those of standards using Empower software (Waters, Corp., Milford, MA, United States).

#### 2.5.3 Fatty acid profiling

Fatty acid extraction was performed according to Folch method (41) with minor modifications. Approximately 800–1,000 mg of dried *Rhodotorula* biomass was homogenized with 50 mL of chloroform:methanol (2:1, v/v) and 10 mL of distilled water. The lower chloroform layer was collected and filtered through anhydrous sodium sulfate. The chloroform was then concentrated under reduced pressure at 40 °C using a rotary evaporator (RE-100-Pro, DLAB Scientific Co. Ltd., China) and stored under nitrogen at 4 °C until analysis. For methyl ester synthesis, 5 mL of 0.5 N methanolic KOH was added to the concentrate and refluxed for 20 min at 40–50 °C followed by the addition of 5 mL of methanolic boron trifluoride reagent. After cooling, petroleum ether and saturated NaCl solution were added to facilitate phase separation. The organic layer was collected, passed through anhydrous sodium sulfate, and concentrated under nitrogen. The fatty acid methyl esters (FAMEs) were dissolved in a 1 mL hexane and stored in amber vials under nitrogen until gas chromatographic (GC) analysis. GC was performed using a Thermo Scientific TRACE™ 1,110 GC system equipped with a flame ionization detector. Ultra-high purity hydrogen and nitrogen gases (99.9995%) were used as carrier and makeup gases, respectively. The analysis was conducted on a TR-FAME column (30 m × 0.25 mm i.d., 0.25 μm film thickness) with a column temperature of 240 °C, injection volume of 2 μL, and a flow rate of 1.2 mL min^−1^. Fatty acid methyl esters were identified by comparing their retention times with those of standard FAME mixtures.

### 2.6 Statistical analysis

The statistical analysis was performed using R version 4.5.1. Data were checked for normality and homoscedasticity by Shapiro–Wilk and Bartlett’s test, respectively. Parametric one-way ANOVA was performed when the assumptions of ANOVA were satisfied. In the case of non-parametric data, statistical differences were evaluated using the Kruskal-Wallis test. For parametric datasets, post-hoc comparisons were performed using Duncan’s multiple range test (*α* = 0.05). For non-parametric datasets, Dunn’s test was applied. All experiments were conducted in triplicates. Results are expressed as mean ± standard error, with a significance level set at *p* < 0.05. R Package *ggplot2* was used for data visualization (42).

## 3 Results

### 3.1 Preliminary screening and isolation

After 2–3 days of culture, colonies appeared at the interface of the leaf material and agar (Supplementary Figure 1B), indicating the successful emergence and growth of endophytic microorganisms from the plant tissue onto the culture medium. Colonies were creamy white and gradually turned pale orange over the following days of culture (Supplementary Figure 1C). The isolate formed creamy, circular colonies on sheep blood agar, with no detectable *α*- or *β*-hemolysis, (Supplementary Figure 2A). With regards to extracellular enzyme production capacity, the strain did not show any halo or clearance zone formation for phospholipase, lipase and proteinase tests (Supplementary Figures 2B–D).

### 3.2 Growth performance and media optimization

**Figure 2:**
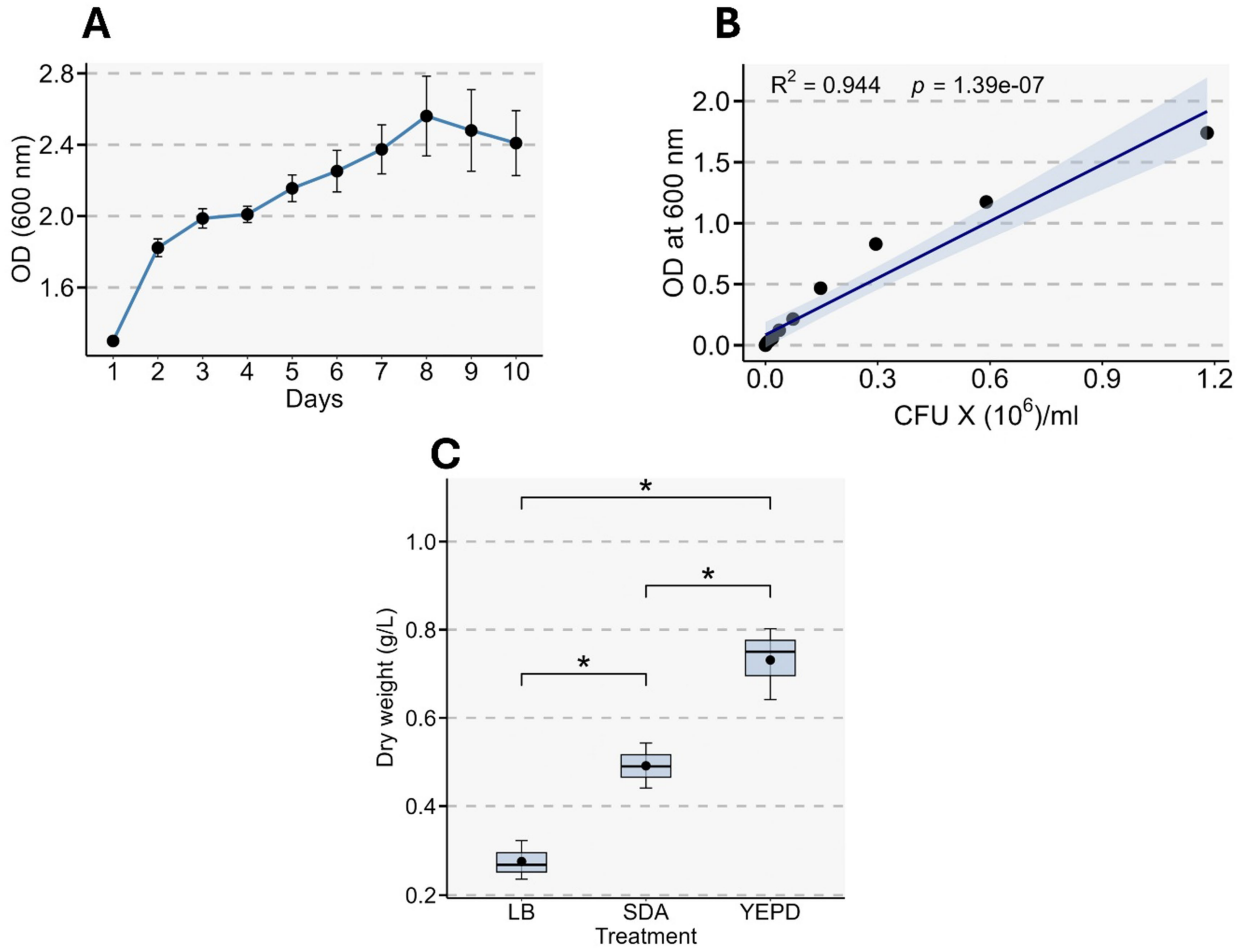
Growth characterization and biomass comparison across culture media. **(A)** Optical density (OD_600_) measurements over a 10 day period showing the growth kinetics of the culture. Points represent mean ± SE of three biological replicates. **(B)** Linear regression of OD_600_ versus colony-forming units (CFU), demonstrating a strong positive correlation between optical density and viable cell concentration (*R*^2^ = 0.944, *p* = 1.39 × 10^−7^). Shaded region indicates the 95% confidence interval of the regression. **(C)** Boxplots showing dry biomass yield (g L^−1^) across three different media (LB, SDA, YEPD). Statistical differences among treatments were evaluated using one-way ANOVA followed by post-hoc testing. The black dot in each box represents the mean value of three biological replicates. * indicates *p* < 0.05.

The OD of the *Rhodotorula* culture was monitored to assess growth (Figure 2A). The culture showed a typical microbial growth pattern, starting with an initial lag phase, followed by an exponential growth phase that lasted 2 days. A stationary phase was observed thereafter with maximum biomass on the 8th day, followed by a decline. A strong correlation was observed between OD and CFU (R^2^ = 0.944, *p* < 0.0001) (Figure 2B). The biomass accumulation of the microbe in YEPD was significantly higher than in SDA or LB broth (Figure 2C).

### 3.3 Microscopy

**Figure 3:**
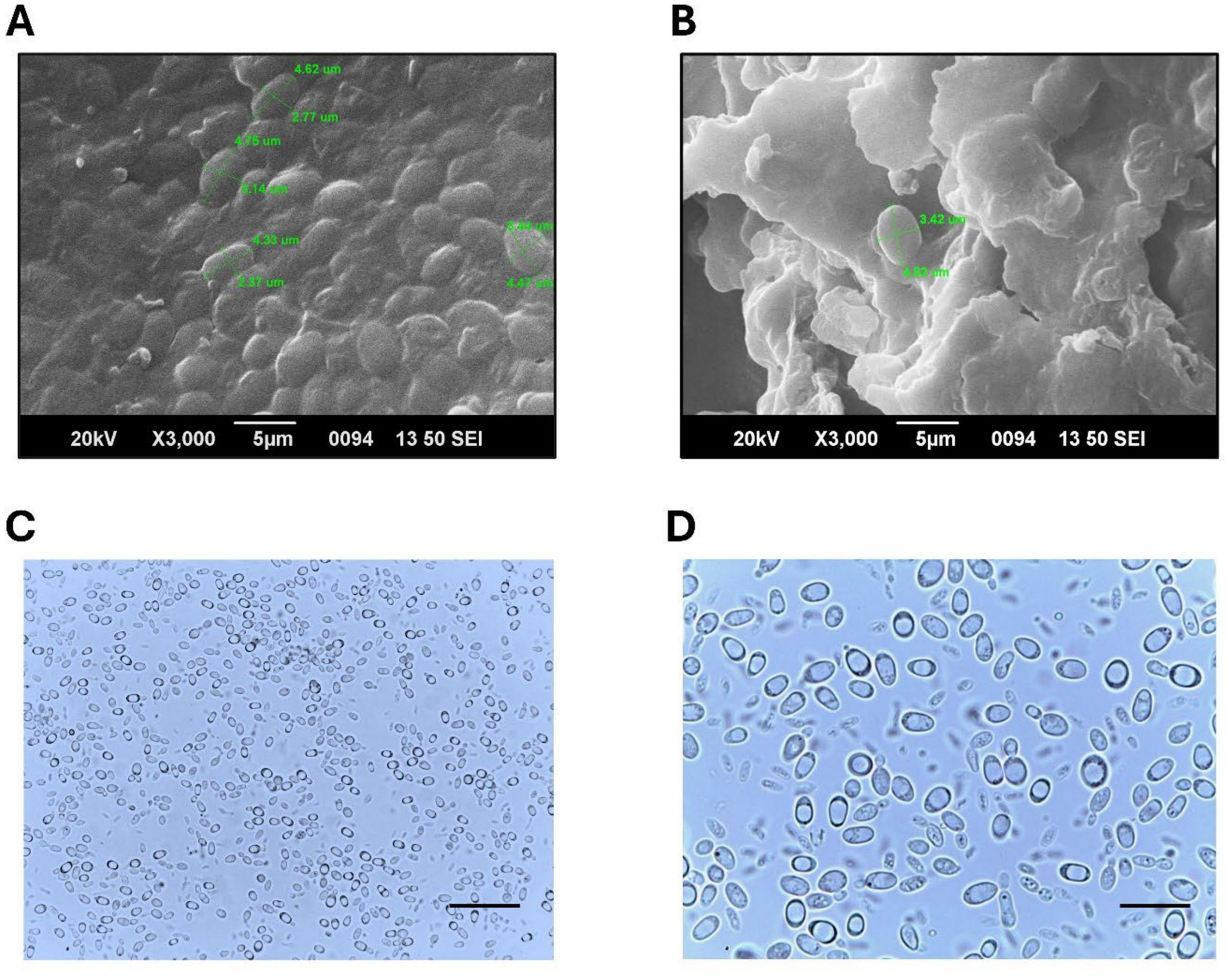
Morphological characterization of *Rhodotorula* sp. using scanning electron microscopy (SEM) and light microscopy. **(A)** SEM micrograph showing the surface morphology of freeze dried *Rhodotorula* biomass at 3000 × magnification. Scale bar = 5 μm. **(B)** SEM image highlighting larger intact cells with diameters of approximately 3–4 μm. Scale bar = 5 μm. **(C)** Light microscopy image of *Rhodotorula* cells under brightfield illumination at a magnification of 400×, scale bar = 25 μm. **(D)** Light microscopy image of *Rhodotorula* cells under brightfield illumination at a magnification of 1,000×, scale bar = 10 μm.

The morphology of *Rhodotorula* sp. cells was examined using both SEM and light microscopy. The SEM analysis revealed oval cells with size approximately 3.4 to 4.8 μm in diameter (Figures 3A,B). Light micrographs further confirmed the oval morphology of the *Rhodotorula* sp. Budding cells typically produced a single daughter cell, although two daughter cells occasionally emerged from the same budding site. Large non-dividing cells showed prominent vacuolization occupying much of the cytoplasm (Figures 3C,D). Overall, the cells appeared uniformly distributed with no hypal or pseudohyphael formations.

### 3.4 18S rRNA and ITS gene sequencing and MALDI TOF mass spectroscopy

**Figure 4:**
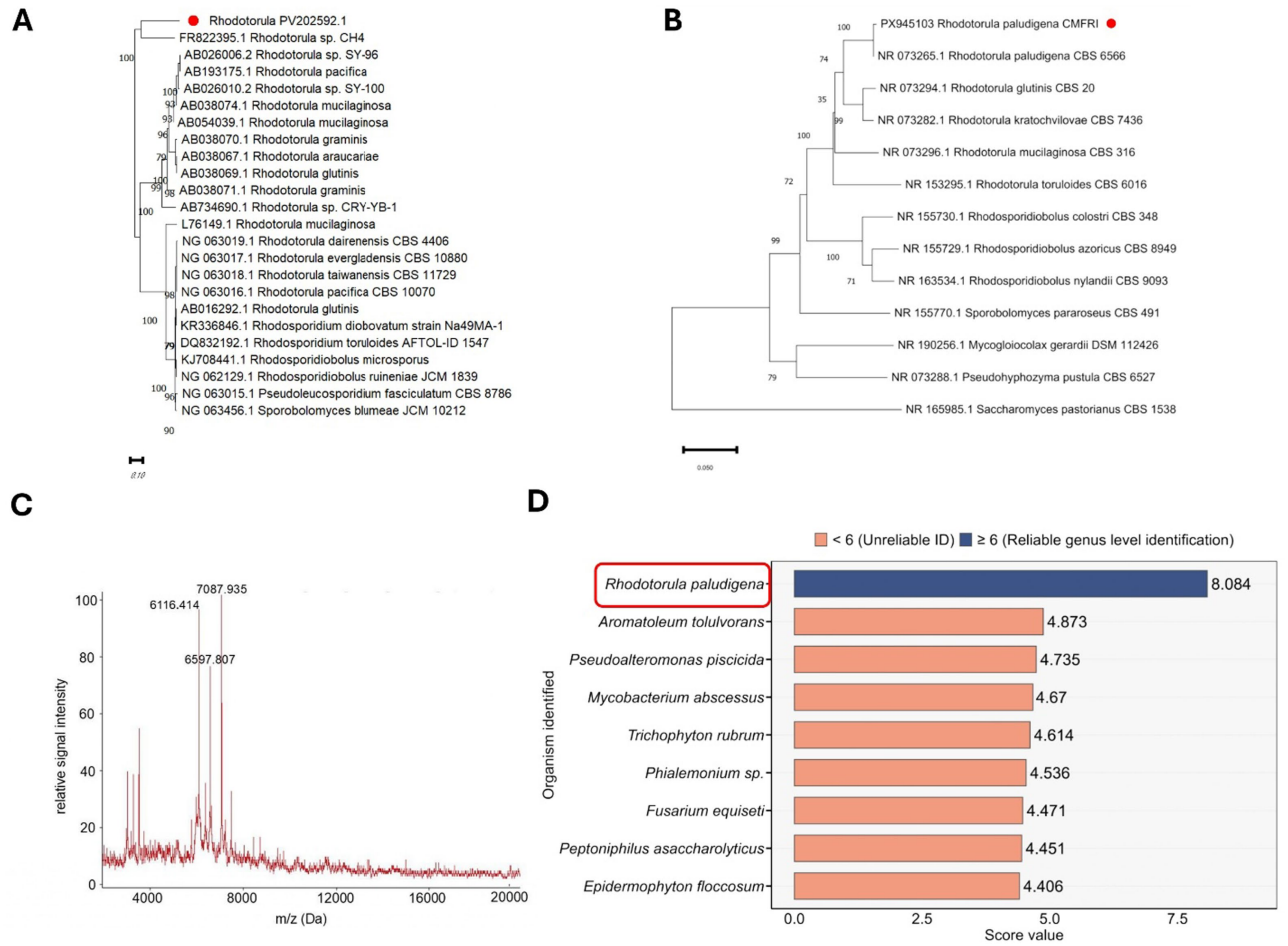
Phylogenetic analysis and MALDI-TOF MS-based identification of *Rhodotorula* sp. isolate. **(A)** Neighbor-joining phylogenetic tree based on the 18S rRNA gene sequence showing the relationship of the isolate with other *Rhodotorula* species. The isolate clustered closely with *Rhodotorula* sp. CH4 (FR822935.1) with strong bootstrap support (>90%), confirming its placement within the *Rhodotorula* clade. **(B)** Neighbor-joining phylogenetic tree based on the ITS gene sequence showing the relationship of the isolate with other *Rhodotorula* species. The isolate clustered with *Rhodotorula paludigena*. **(C)** Representative MALDI-TOF MS spectrum of the isolate showing major protein peaks at 6116.414, 6637.807, and 7087.935 m/z. **(D)** Comparative MALDI-TOF Biotyper score values for the ten closest matches. *Rhodotorula paludigena* showed the highest score (8.084), exceeding the reliability threshold (score ≥ 6.0) for genus-level identification. Bar in blue indicate reliable genus-level identifications, while orange bars represent unreliable matches (score < 6.0).

The phylogenetic analysis of the 18S rRNA gene sequence revealed that the isolate was placed within the *Rhodotorula* clade containing *R. mucilaginosa*, *R. graminis* and *R. pacifica* etc. (Figure 4A). The phylogenetic tree further revealed that the isolate clustered closely with *Rhodotorula* sp. CH4 (accession no. FR822935.1). The ITS sequencing revealed that the sequence was a 99.82% identity (Figure 4B) with *R. paludigena* (accession no. NR_073265.1). The 18S rRNA and ITS gene sequences of the isolate are submitted in NCBI database (accession numbers PV202590 and PX945103, respectively). The MALDI-TOF mass spectrometry yielded a distinct spectral fingerprint for the isolate with three major peaks at 6116.414, 6637.807, and 7087.935 m/z (Figure 4C). Comparing this fingerprint with the reference database revealed again the highest log score of 8.04 for *Rhodotorula paludigena* (Figure 4D).

### 3.5 Pigment characterization and quantification

**Figure 5:**
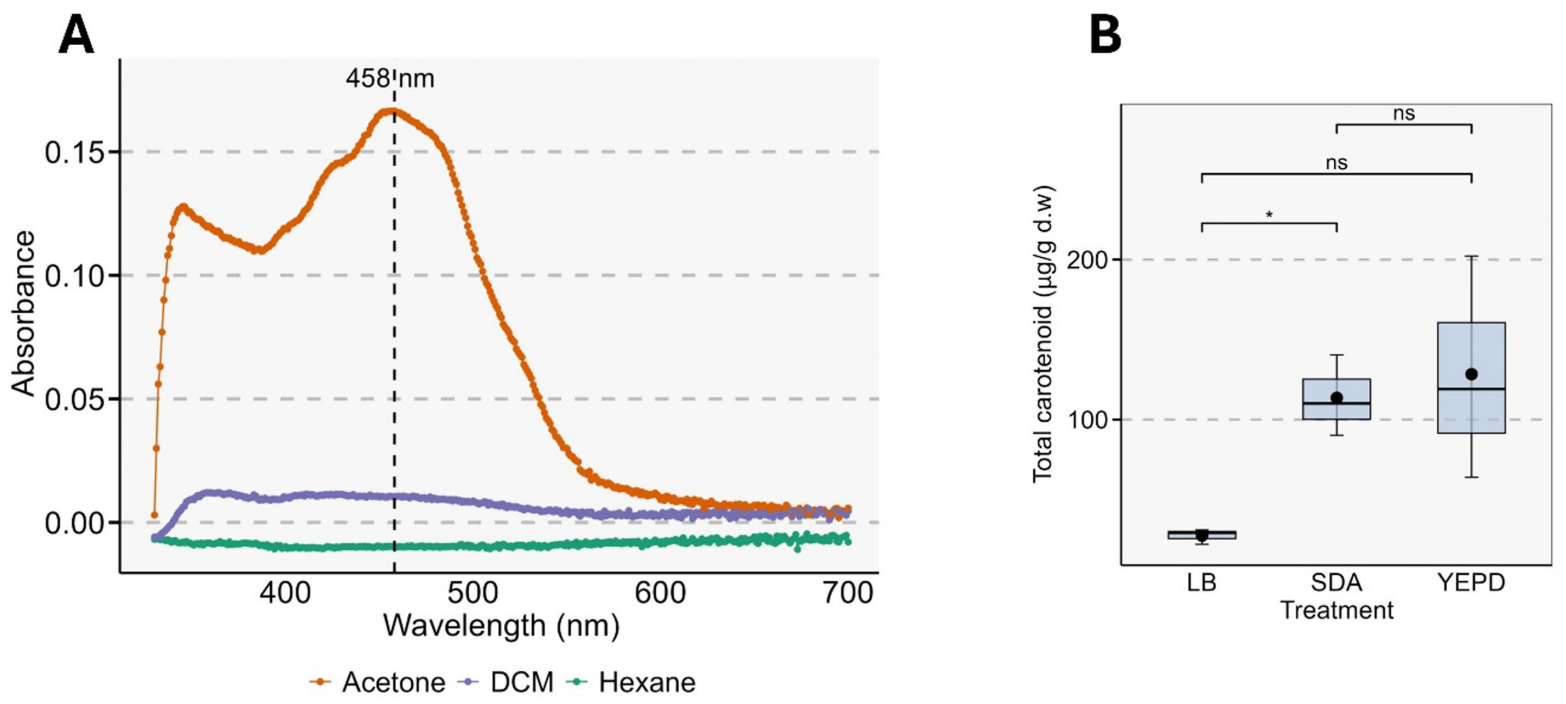
Carotenoid detection, extraction characteristics, and quantification in *Rhodotorula* sp. **(A)** UV–visible absorbance spectra of carotenoid extract obtained using acetone, dichloromethane, and hexane as solvents. The acetone extract displays a distinct carotenoid peak at 458 nm, characteristic of *Rhodotorula*-derived pigments such as torulene and β-carotene. **(B)** Boxplots showing total carotenoid content (μg g^−1^ dry weight) of cultures grown in LB, SDA, and YEPD media. Statistical comparisons were performed using one-way ANOVA followed by post-hoc testing. The black dot represents the mean value for each treatment. In the plot, * indicates *p* < 0.05 and ns indicates no significant difference.

Of the three solvents used to extract the pigments from *Rhodotorula*, acetone showed the highest solubilization efficiency. The spectroscopic scan of the crude acetone extract revealed a broad but single major peak at 458 nm, whereas n-hexane and DCM showed no detectable peaks (Figure 5A). Pigment production of *Rhodotorula* also varied significantly among the culture media. YEPD supported the highest pigment accumulation (128.38 ± 40.2 μg/g dry weight), followed by SDA (113.57 ± 14.61 μg/g) which was significantly higher than LB medium (27.43 ± 2.72 μg/g) (Figure 5B).

**Figure 6:**
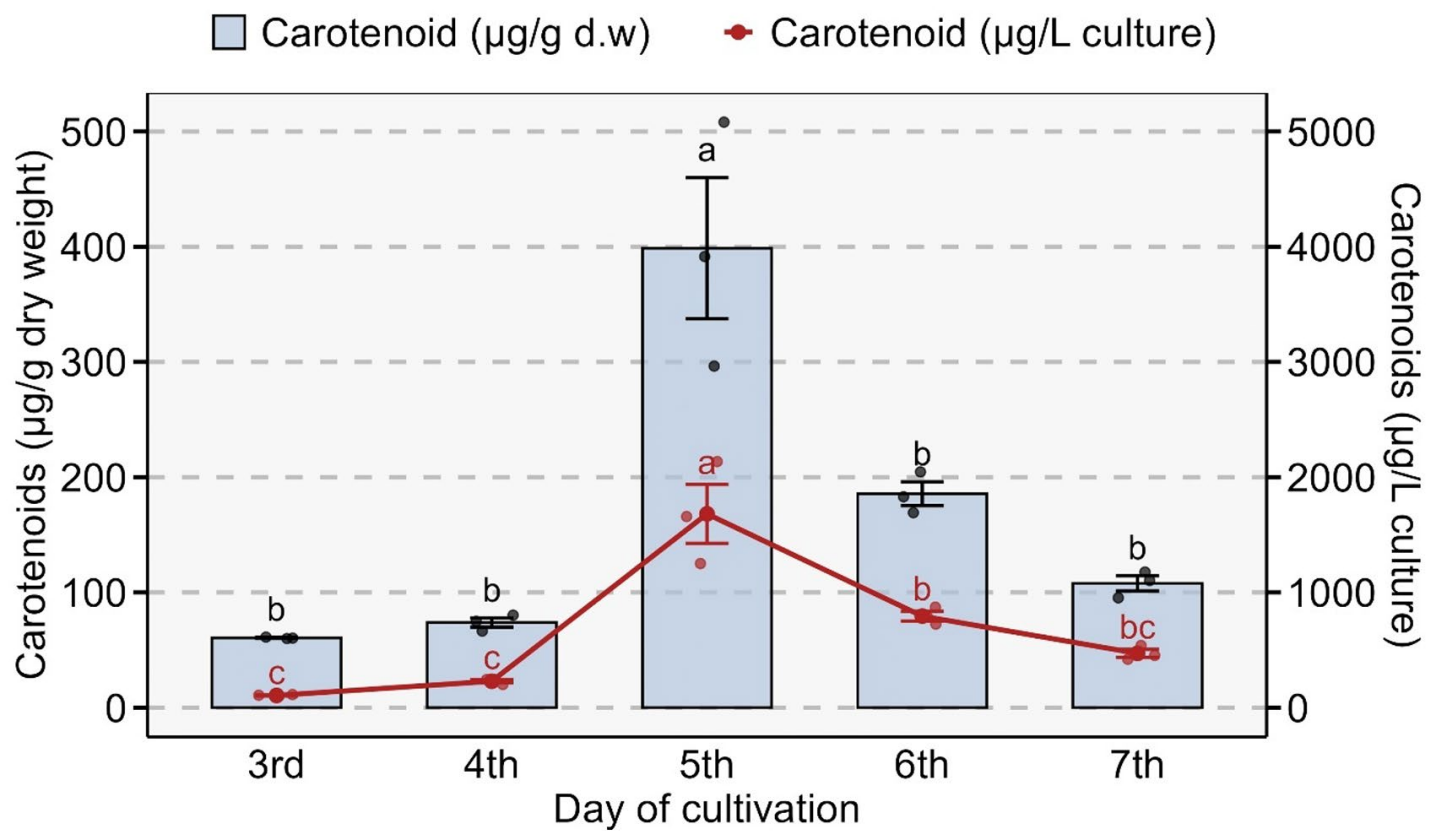
Carotenoid production profile of *Rhodotorula* sp. across different cultivation days. Bar plots represent intracellular carotenoid content (μg g^−1^ dry weight), while the red line shows total carotenoids normalized to culture volume (μg L^−1^). Maximum intracellular carotenoid accumulation occurred on the 5th day of cultivation, followed by a decline in subsequent days. Different letters above bars indicate statistically significant differences (*p* < 0.05) based on one-way ANOVA followed by post-hoc testing. Error bars represent standard deviation of biological replicates.

Pigment synthesized by *Rhodotorula* sp. also varied markedly with the cultivation period. Pigment content increased from 3rd day of culture to day 5 peaking at 398 ± 61.2 μg/g dry weight (1,681 ± 255.6 μg/L culture). After day 5, pigment levels declined gradually to 107 ± 6.5 μg/g dry weight (470 ± 35.3 μg/L culture) (Figure 6).

### 3.6 Antioxidant potential

**Figure 7:**
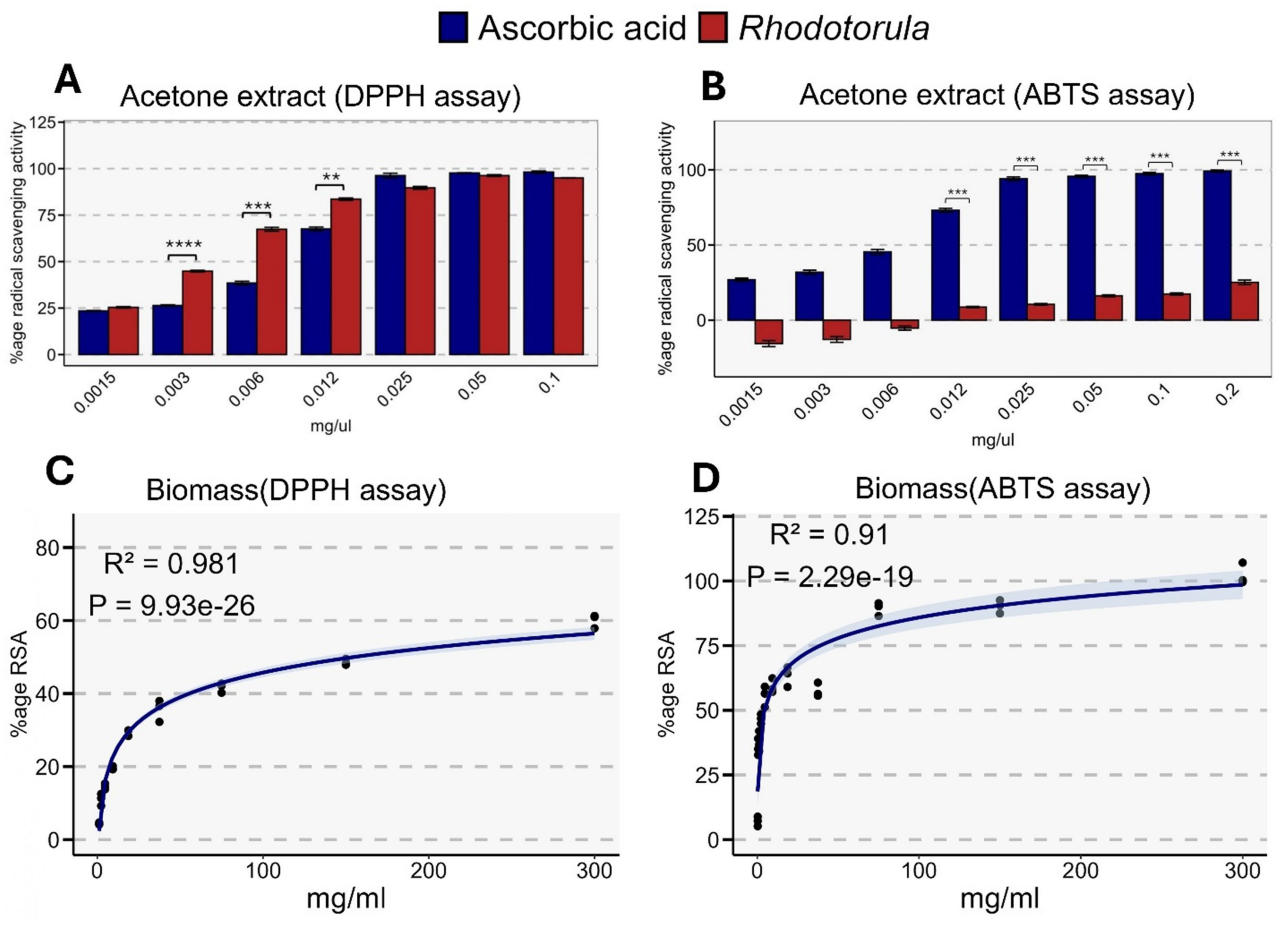
Antioxidant activity of *Rhodotorula* sp. acetone extracts and biomass evaluated using DPPH and ABTS radical scavenging assays. **(A)** DPPH radical scavenging activity of acetone extracts across increasing concentrations (mg μL^−1^), compared with ascorbic acid as a positive control. *Rhodotorula* extracts show a dose-dependent increase in antioxidant activity, with significant differences from ascorbic acid at lower concentrations. **(B)** ABTS radical scavenging activity of acetone extracts, again benchmarked against ascorbic acid. Ascorbic acid exhibits consistently higher scavenging efficiency, while *Rhodotorula* extracts show modest but measurable activity. **(C)** Dose–response curve for biomass-based DPPH activity, showing a strong nonlinear relationship between biomass concentration and % radical scavenging activity (RSA). Model fit statistics: R^2^ = 0.981, *p* = 9.93 × 10^−26^. **(D)** Biomass-based ABTS activity showing similar nonlinear behavior, with increasing RSA at higher biomass concentrations (R^2^ = 0.91, *p* = 2.29 × 10^−19^). Shaded regions in **(C,D)** represent the 95% confidence interval of the fitted curve. Statistical significance in **(A,B)** is indicated as follows: *p* < 0.05, *p* < 0.01, *p* < 0.001, *p* < 0.0001.

The antioxidant potential of the crude acetone extract of *Rhodotorula* sp. was assessed using the DPPH and ABTS radical scavenging assays. When compared to the ascorbic acid standard, the crude acetone extract of *Rhodotorula* demonstrated comparable antioxidant activity across concentrations ranging from 0.025 to 1 mg/mL (Figure 7A). In contrast, the ABTS assay showed significantly lower antioxidant capacity than the ascorbic acid standard across concentration range of 0.012 to 0.2 mg/mL (Figure 7B). Additionally, the radical scavenging activity of the whole lyophilized biomass of *Rhodotorula* sp. was assessed using both DPPH and ABTS assays. In both assays, and radical scavenging activity increased strongly with biomass concentration (R^2^ = 0.98, *p* < 0.0001 for DPPH; R^2^ = 0.91, *p* < 0.0001 for ABTS) (Figures 7C,D). At 300 mg/mL, radical scavenging activity reached 60.03 ± 1.07% in the DPPH assay and 96.34 ± 5.37% in the ABTS assay.

### 3.7 Antimicrobial and antibiofilm activity

**Figure 8:**
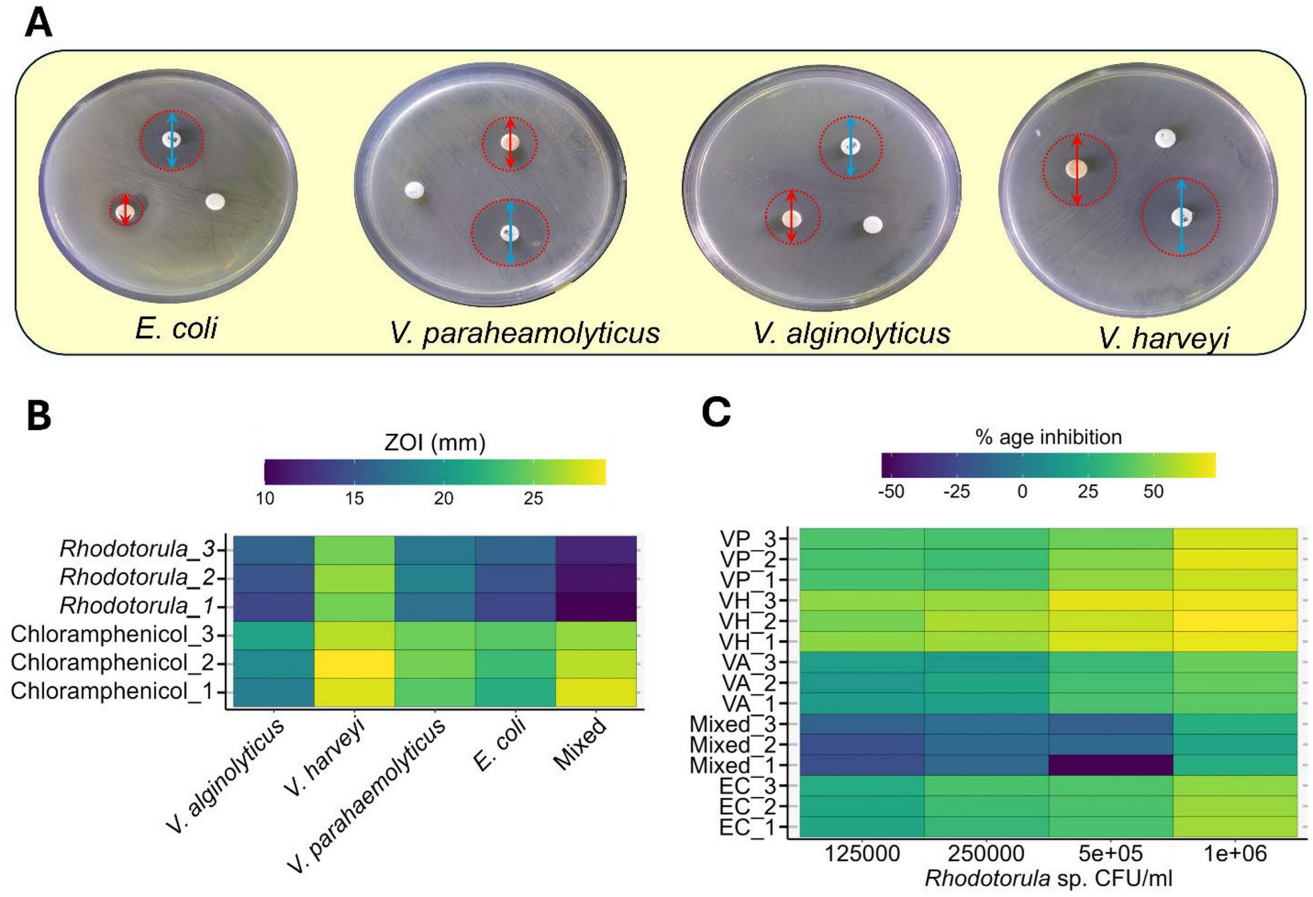
Antibacterial activity of *Rhodotorula* sp. against selected gram-negative bacteria. **(A)** Representative agar diffusion plates showing zones of inhibition (ZOI) produced by *Rhodotorula* sp. biomass or extract against *E. coli*, *Vibrio parahaemolyticus*, *Vibrio alginolyticus*, and *Vibrio harveyi*. The white discs represent controls, while red and blue arrows indicate the zones produced by *Rhodotorula* samples and antibiotic controls, respectively. **(B)** Heatmap visualizing ZOI values (mm) generated by three biological replicates of *Rhodotorula* sp. and chloramphenicol controls against each bacterial species. Higher ZOI values (yellow) indicate stronger antibacterial activity, while lower values (purple) indicate minimal inhibition. **(C)** Inhibition efficiency (%) across varying concentrations of *Rhodotorula* sp. (CFU mL^−1^) against each test organism. Heatmap shading represents percent inhibition relative to untreated controls, with positive values indicating growth suppression and negative values indicating growth promotion or no inhibition. VP: *V. parahaemolyticus*; VH: *V. harveyi*; VA: *V. alginolyticus*; EC: *E. coli*.

The ability of *Rhodotorula* sp. to inhibit the growth of pathogenic bacteria, including *Vibrio parahaemolyticus*, *V. alginolyticus*, *V. harveyi* and *Escherichia coli*, was evaluated using the agar well diffusion method (Figure 8A). The crude acetone extract of *Rhodotorula* was effective against all four pathogens, with zones of inhibition of 17.66 ± 0.44, 15.00 ± 0.57, 25.30 ± 0.33 and 15.00 ± 0.57 mm for *V. parahaemolyticus*, *V. alginolyticus*, *V. harveyi* and *E. coli*, respectively (Figure 8B). Notably, the inhibition of *V. harveyi* was the highest among the tested bacteria, although it remained significantly lower than that achieved by chloramphenicol (25.3 ± 0.33 mm for *Rhodotorula* extract vs. 28.0 ± 0.57 mm for chloramphenicol; *p* = 0.02). The crude acetone extract showed multiple zones of inhibition in the mixed culture plate (Supplementary Figure 3).

Antibiofilm activity of *Rhodotorula* sp. against pathogenic bacteria was evaluated at four different concentrations of 125,000, 250,000, 500,000 and 1,000,000 CFU/mL of the isolate (Figure 8C). The percentage antibiofilm activity increased with increasing CFU of *Rhodotorula* sp. against all four pathogens tested. Maximum inhibition occurred against *Vibrio harveyi* (71.12 ± 1.16% inhibition), followed *V. parahaemolyticus* (65.70 ± 1.3% inhibition), *E. coli* (54.08 ± 0.9% inhibition) and *V. aginolyticus* (43.06 ± 0.81% inhibition).

### 3.8 Thermal resistance, simulated gastric and intestinal fluid challenge

**Figure 9:**
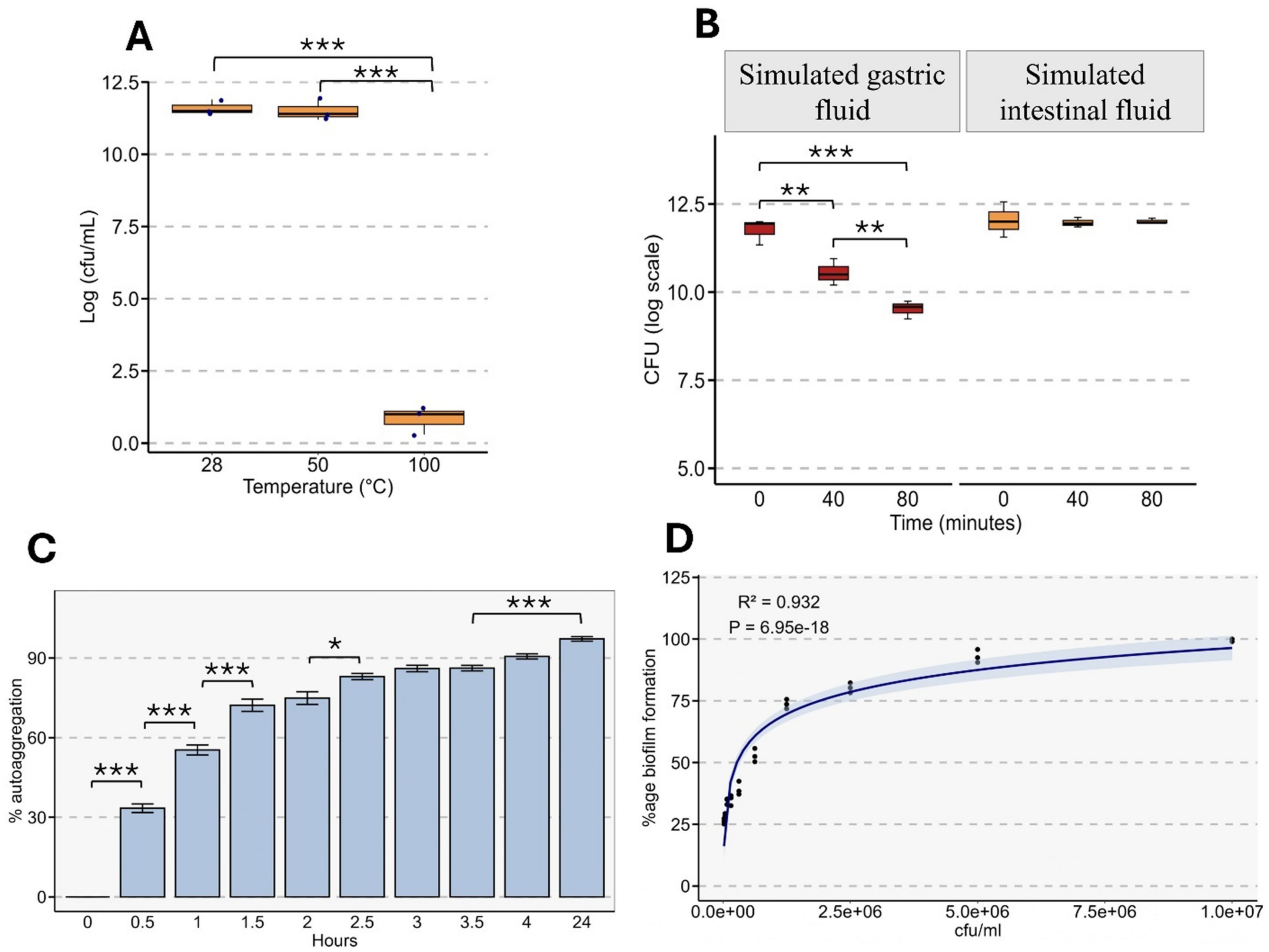
Stress tolerance, aggregation, and biofilm-forming properties of *Rhodotorula* sp. **(A)** Thermal tolerance of *Rhodotorula* sp., shown as viable cell counts (log CFU mL^−1^) after exposure to 28 °C, 50 °C, and 100 °C. Significant reductions in viability occur at elevated temperatures, particularly at 100 °C. **(B)** Survival of *Rhodotorula* sp. in simulated gastric fluid (SGF; pH ≈ 2.0) and simulated intestinal fluid (SIF; pH ≈ 7.5) at 0, 40, and 80 min. A marked decline in CFU is observed under SGF conditions, while viability remains stable in SIF. **(C)** Autoaggregation ability of *Rhodotorula* sp. over a 24-h period. Aggregation increases steadily with time, reaching its highest level at 24 h. Significant differences across time points are indicated. **(D)** Biofilm formation as a function of inoculum concentration (CFU mL^−1^). A nonlinear regression model shows a strong positive correlation between cell density and biofilm biomass (*R*^2^ = 0.932, *p* = 6.95 × 10^−18^). Shaded region indicates the 95% confidence interval of the fitted curve. Statistical significance: *p* < 0.05, *p* < 0.01, *p* < 0.001.

The viability of *Rhodotorula* sp. was evaluated under different temperatures and in simulated gastrointestinal conditions. Exposure to 50 °C caused a no significant reduction in cell viability compared with the control at 28 °C (11.60 ± 0.15 to 11.50 ± 0.20 Log₁₀ CFU/mL; *p* > 0.05). In contrast, incubation at 100 °C caused a severe decline in viability, indicating sensitivity to extreme heat (0.833 ± 0.27 Log₁₀ CFU/mL; *p* < 0.001) (Figure 9A).

In simulated gastric fluid, viability of *Rhodotorula* sp. decreased significantly after 30 min, dropping from 11.76 ± 0.28 to 10.54 ± 0.21 Log₁₀ CFU/mL (*p* < 0.01). After 80 min, viability declined further to 9.52 ± 0.14 Log₁₀ CFU/mL (*p* < 0.001 vs. control), indicating reduced stability over time (Figure 9B). Conversely, incubation in simulated intestinal fluid did not significantly impact the viability, with only minimal changes observed after 40 min (12.04 ± 0.28 to 11.97 ± 0.07 Log₁₀ CFU/mL; *p* > 0.05) and 80 min (12.04 ± 0.28 to 12.01 ± 0.04 Log₁₀ CFU/mL; *p* > 0.05).

### 3.9 Autoaggregation and biofilm formation

Autoaggregation of *Rhodotorula* sp. was evaluated over 24 h to assess its ability to self- adhere without involvement of other microbial species, a key probiotic trait. Within 30 min, the percentage autoaggregation increased from 0 to > 30% (*p* < 0.001) (Figure 9C). Autoaggregation continued to increase consistently up to 24 h reaching > 90% (*p* < 0.001). Biofilm formation was evaluated at microbial loads ranging from 2 × 10^4^ to 1 × 10^7^ CFU/mL. Biofilm formation was strongly correlated with the cell concentration (R^2^ = 0.932, *p* < 0.001) reaching 99.66 ± 0.33% at 1 × 10^7^ CFU/mL (Figure 9D).

### 3.10 Nutritional profiling of *Rhodotorula* sp.

**Figure 10:**
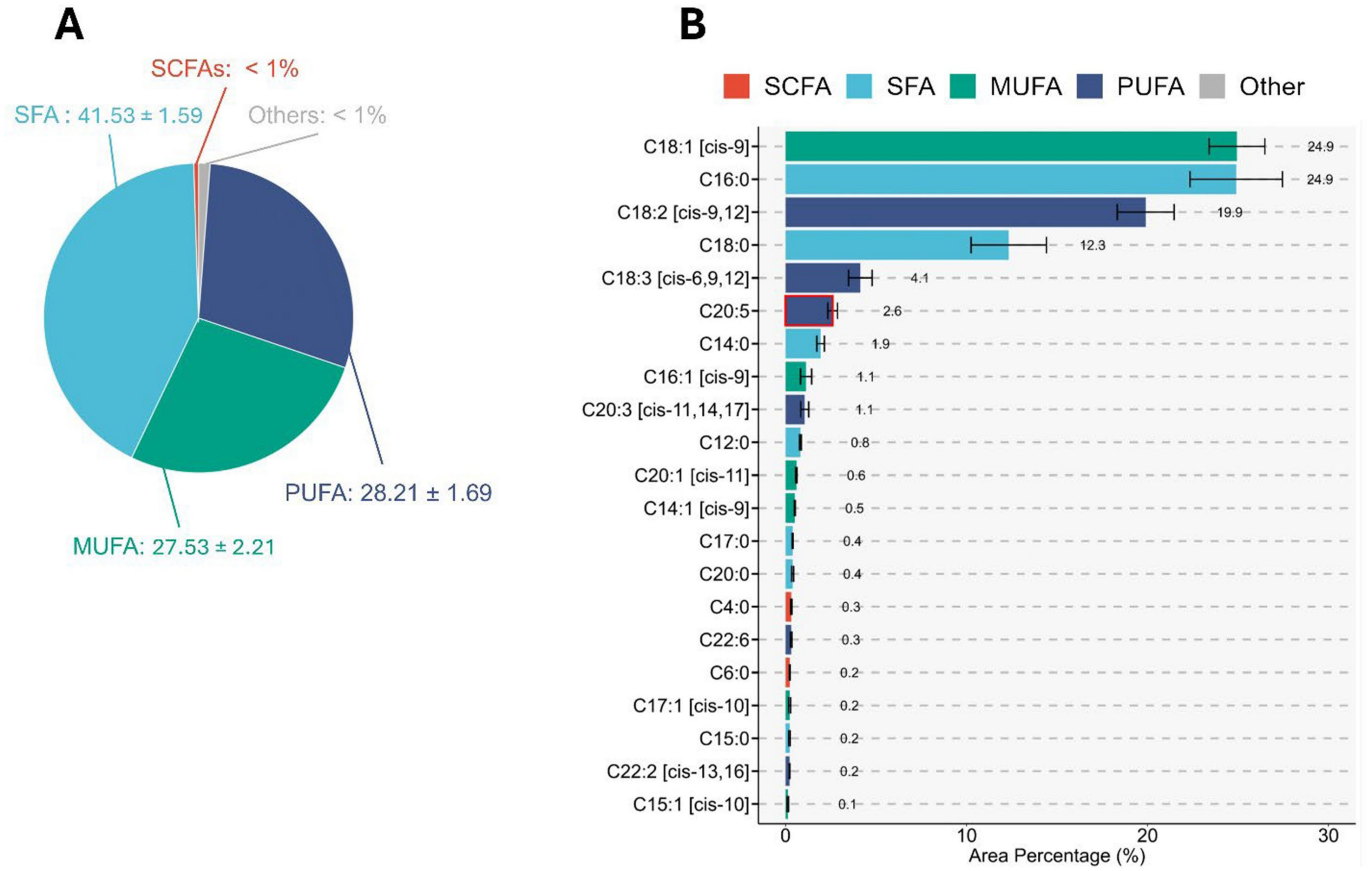
Fatty acid composition of *Rhodotorula* sp. biomass. **(A)** Proportional distribution of major fatty acid groups based on total lipid extracts. Saturated fatty acids (SFA) constitute the largest fraction (41.53 ± 1.59%), followed by monounsaturated fatty acids (MUFA; 27.53 ± 2.21%) and polyunsaturated fatty acids (PUFA; 28.21 ± 1.69%). Short-chain fatty acids (SCFA) and other minor components each account for <1% of the total fatty acid profile. **(B)** Detailed fatty acid composition presented as area percentage of individual fatty acids. MUFAs (particularly C18:1 and C16:0) dominate the profile, with notable contributions from PUFAs such as C18:2 and C18:3. Trace levels of SCFAs and other minor fatty acids are also detected. Fatty acid classes are color-coded as SCFA, SFA, MUFA, PUFA, and other components for clarity. Error bars represent standard deviations from biological replicates.

**Figure 11:**
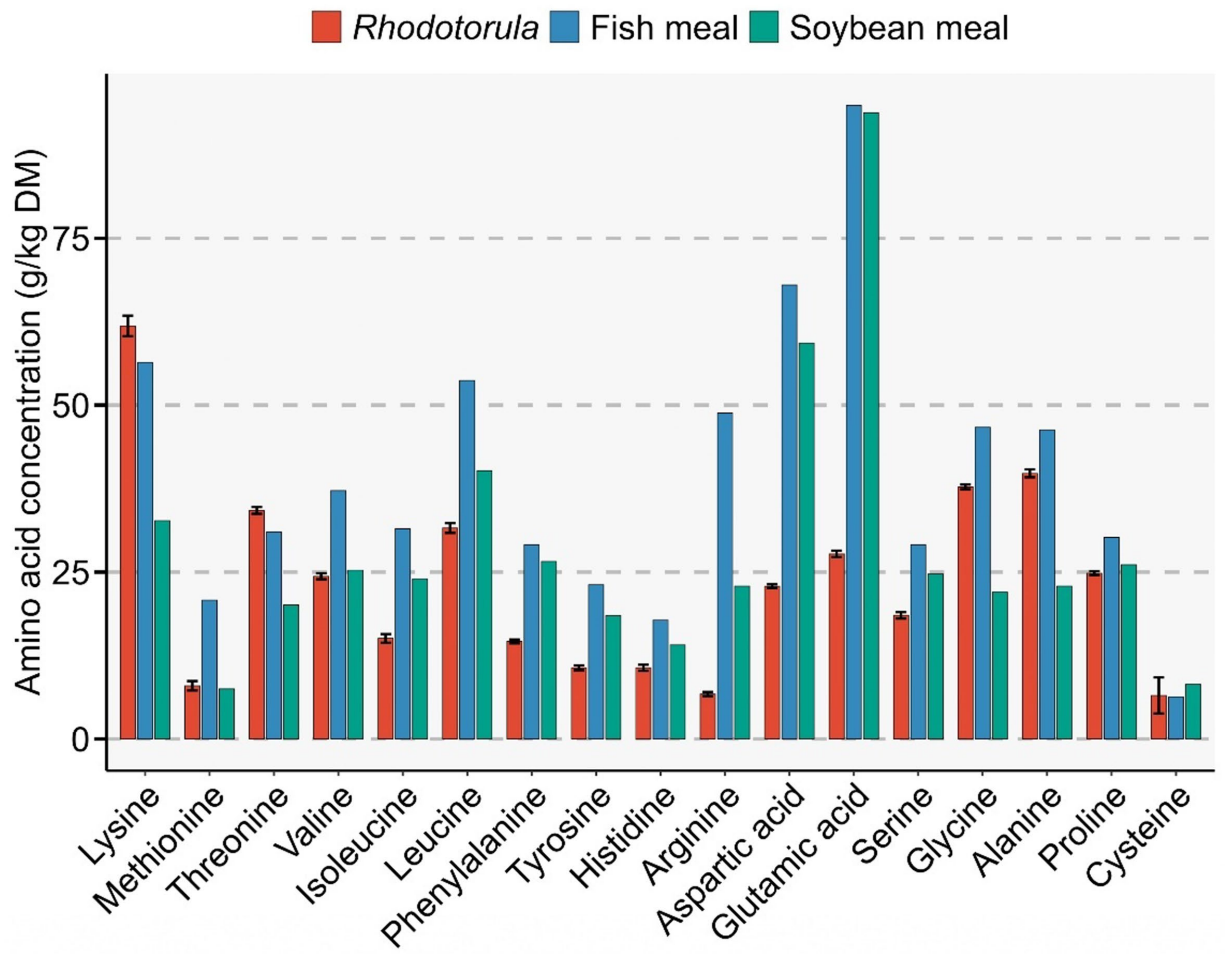
Comparative amino acid profile of *Rhodotorula* sp. biomass, fish meal, and soybean meal. Bar plots show the concentration of essential and non-essential amino acids (g kg^−1^ dry matter) across the three protein sources. *Rhodotorula* sp. displays appreciable levels of several essential amino acids, including lysine, methionine, threonine, valine, and leucine, though generally lower than fish meal and soybean meal. Notably, soybean meal exhibits the highest concentrations of glutamic acid and serine, while fish meal shows elevated levels of lysine and histidine. Error bars for *Rhodotorula* represent standard deviations from biological replicates. The amino acid profile of fish meal and soybean meal was obtained from FeedTables website (https://www.feedtables.com/content/soybean-meal-oil-5-48-protein-oil; https://www.feedtables.com/content/fish-meal-protein-70).

The crude protein, ash and crude lipid content of *Rhodotorula* were 40 ± 2.4%, 3 ± 0.5% and 2 ± 0.2%, respectively. The fatty acid profile showed that PUFA represented 28.21 ± 1.69% of the total fatty acids, whereas MUFA and SFA accounted for 27.53 ± 2.21 and 41.53 ± 1.59%, respectively (Figure 10A). Oleic acid (C18:1 [cis-9]), Palmitic acid (C16:0) and Linoleic acid (C18:2 [cis-9,12]) were the most abundant MUFA, SFA and PUFA occurring at 24.9 ± 0.8, 24.9 ± 1.4 and 19.9 ± 0.90% of total fatty acids, respectively. Docosahexaenoic acid (DHA) and eicosapentaenoic acid (EPA) accounted for 0.3 ± 0.02 and 2.6 ± 0.15% of the total fatty acids, respectively (Figure 10B). Lysine was the most abundant amino acid in *Rhodotorula.* The concentration of lysine (61.86 ± 1.53 g/kg dry matter) and threonine (34.23 ± 0.51 g/kg) were higher than those in both fish meal and soybean meal. In *Rhodotorula*, the amount of alanine (39.78 ± 0.57 g/kg) and glycine (37.76 ± 0.34 g/kg) were present at higher concentrations than in soybean meal but lower than in fish meal (Figure 11).

## 4 Discussion

Mangrove ecosystems are among the most productive coastal habitats, functioning as nurseries for marine organisms and as natural buffers that protect shorelines from erosion. These habitats also sustain diverse microbial communities, including bacteria, protists, and fungi, many of which possess nutritional, therapeutic, and biotechnological potential. The pigment-containing yeast *Rhodotorula* spp. have been investigated for various biotechnological purposes that include wastewater pollutant breakdown (43), antimicrobial potential (44), pigment production (45), and immune stimulation (46). In addition, yeasts of the genus *Rhodotorula* have gained attention for their potential probiotic properties, particularly in aquaculture and functional nutrition. Certain *Rhodotorula* strains produce carotenoid pigments such as *β*-carotene, torulene, and torularhodin, which exhibit strong antioxidant activity and can contribute to host health (47). In addition, non-*Saccharomyces* yeasts, including *Rhodotorula*, have been reported to enhance immune responses, improve gut microbial balance, and exert antagonistic effects against pathogenic microorganisms, thereby supporting growth performance and disease resistance in aquatic organisms (48). These functional attributes highlight their potential as emerging probiotic candidates. However, their application requires careful strain-specific evaluation, as some *Rhodotorula* species have also been described as opportunistic pathogens in immunocompromised individuals (49). In this study, we isolated and characterized a novel pigment-containing *Rhodotorula* sp. Because *in vivo* trials with fish, for assessment of potential probiotic functions, are expensive and often influenced by host-specific factors and gut microbiome variability (50), we performed a polyphasic *in vitro* evaluation to determine whether probiotic potential exists in the isolated strain or not. The polyphasic approach included pigment production, antimicrobial potential, and viability in the simulated gut conditions as some of the key parameters. This strategy combined morphological, biochemical, molecular, and functional assays on a *Rhodotorula* isolate from mangrove ecosystem.

The phylogenetic analysis of the 18S rRNA gene from the isolate indicated that it clusters closely to *Rhodotorula* sp. CH4. The *Rhodotorula* sp. CH4 is a strain of *Rhodotorula mucilaginosa* that has shown potential for degrading the phenolic pollutants like protocatechuic acid, vanillic acid, p-coumaric acids, and tyrosol that are released from olive mill wastewater (43). This species is also known to synthesize pigments, lipids and enzymes and is regarded as an excellent biorefinery for bioactive compounds (51). *R. mucilaginosa* has also shown immunomodulatory effects in mice as oral gavage of this probiotic causes an increase in the thymus and spleen size and increase in the circulating IgG, IgA and IL-2 levels (52). In contrast, the ITS gene sequencing and MALDI-TOF MS profile of the current isolate yielded the highest match score for *Rhodotorula paludigena*. Owing to its high pigment-producing capacity, *Rhodotorula paludigena* has also been evaluated for probiotic potential. Feeding *L. vannamei* with a diet containing 5% *R. paludigena* caused an improvement in weight gain and enhanced disease resistance against acute hepatopancreatic necrosis disease caused by *V. parahaemolyticus* (53), with increased expression of immune-responsive genes such as *prophenoloxidase 2* and *lysozyme*, and antioxidant genes including *superoxide dismutase*, *glutathione peroxidase*, and *catalase*. In our study, though both 18S rRNA sequencing and MALDI TOF confirmed the genus of the isolate to be *Rhodotorula*, there were contrasting results with regards to the species level identification. The ITS region is a widely accepted DNA barcode for fungi due to its high interspecific variability and conserved flanking regions, enabling reliable species-level discrimination. ITS sequencing is routinely used for fungal identification and phylogenetic analyses and has been formally recommended as the universal barcode marker for fungi (28). Its effectiveness in distinguishing closely related taxa and its extensive representation in curated databases further support its reliability in taxonomic classification (54). However, a successful identification of a microbe up to species level in MALDI-TOF MS depends on the availability and completeness of high-quality reference spectra (55, 56). Environmental or less-common yeasts, as is the case in the present study, are often underrepresented in the libraries (57, 58). Previous studies have shown that the MALDI-TOF MS database may not reliably distinguish between closely related yeast species, and in such cases, complementary DNA based approach is recommended to ensure accurate taxonomic resolution (59). Nevertheless, the combined phylogenetic and proteomic evidence confirms that the isolate belongs to the genus *Rhodotorula* sp.

Microscopic analysis revealed the *Rhodotorula* sp. isolate as oval cells measuring approximately 3.4 × 4.8 μm. Budding cells were observed singly or in pairs, and the population appeared uniformly distributed. The cells did not present any hyphal or pseudohyphal structures. Though some *Rhodotorula* strains exhibit pseudohyphae development to a limited extent (60) the genus *Rhodotorula* is generally characterized by the absence of pseudohyphae (61). For microscopic investigation, the cells were obtained from the early stationary phase, and as expected considerable number showed monopolar budding. In most cases, a single daughter cell emerged near the budding site as observed by Hamidi et al. (62), and in few instances we found two daughter cells emerging from the budding site. The non-dividing cells exhibited large single vacuoles occupying considerable space in the cytoplasm. This feature of the mature cells is linked to metabolic reorganization, nutrient storage or stress-related response (63).

*Rhodotorula* spp. produce four major carotenoid pigments: *β*-carotene (C_40_H_56_), Torulene (C_40_H_54_), *γ*-carotene (C_40_H_56_), and Torularhodin (C_40_H_52_O_2_) that can co-exist in the yeast with different pigments dominating in different species and with varying culture conditions (64, 65). The appearance of a single, though broad, spectroscopic peak at 458 nm and a single band in TLC indicates one predominant species of pigment in our isolate. This pigment is most likely to be β-carotene as it absorbs light at around 458 nm in acetone (66). Culturing in YEPD media led to the production of the highest amount of pigments compared to SDA and LB agar, in line with previous reports that have confirmed that the absolute and relative amount of each pigment produced by *Rhodotorula* may also vary with media composition (67). The pliability of *Rhodotorula* spp. to modulate their pigment production pathways in response to media and physicochemical conditions forms the basis for industrial production research on this microbe that is focused on the using cheaper media alternatives to reduce the cost of pigment production (67, 68). We found that pigment content varied over time, reaching its maximum on the fifth day of culture. This temporal pigment profile, with a maximum around day 5 of culture, is consistent with previous reports on *Rhodotorula* spp., where carotenoids accumulate mainly during late exponential to stationary phase and often peak between 4 and 7 days of cultivation (69–71).

Though several studies demonstrate that probiotic yeasts exert beneficial effects through antioxidant, anti-inflammatory, and immunomodulatory mechanisms (15, 72, 73), The ability of probiotics to maintain viability under gastric and intestinal conditions is a major challenge for their successful application. We therefore assessed whether the prospective probiotic strain of *Rhodotorula* could withstand harsh gut environments and maintain viability. The isolate did not show any significant decline in the viability in response to incubation in simulated intestinal juice for a duration of 80 min. However, simulated gastric juice markedly diminished the viability after incubation of 40 min that declined further after 80 min of exposure. Similar to our findings, *R. mucilaginosa* TZR_2014_ showed a significant decrease in viability after 30 min of simulated gastric juice exposure (74). These findings corroborate earlier reports that *Rhodotorula* does not survive well in mammalian gastrointestinal tracts (75). Nevertheless, despite its transient presence in the mammalian gut, administration of *Rhodotorula* has consistently produced beneficial immunomodulatory and microbiome-associated outcomes. In mice, dietary supplementation of *Rhodotorula* increased the abundance of beneficial bacterial taxa such as Firmicutes and Lactobacillus, decreased Bacteroidetes, elevated serum IgG, IgA, and IL-2 levels, and enhanced macrophage phagocytosis (52). A likely explanation for the limited natural presence or colonization capacity of *Rhodotorula* in mammals is its relatively low optimum growth temperature (approximately 22–28 °C), which is substantially below the physiological temperatures of mammals (>33 °C). In contrast, fishes are poikilothermic organisms that can have *R. mucilaginosa* as part of their core microbiota, where it exists in a stable synbiotic association with the host (76). Together, these studies indicate that the viability and colonization potential of *Rhodotorula* in vertebrate gastrointestinal systems are highly context dependent and shaped largely by host-specific factors. Nevertheless, evidence from fish suggests a stronger potential of colonization likely favored by temperature and other ecological conditions.

The production of bioactive compounds and colonization resistance against pathogens are two key features of probiotics in aquaculture. Although, exposure to yeast can also influence the gut-associated microbiota profile of fish towards a more robust and disease resistant profile (77, 78), yeast pigments are also biologically significant in the context of probiotic activity. Importantly, yeasts undergo partial cell wall remodeling, autolysis, or phagocytosis in the gut, leading to the release of intracellular metabolites, including lipophilic pigments. Lipophilic intracellular pigments, particularly carotenoids, are widely recognized for their antioxidant, anti-inflammatory, and membrane-protective properties. Several studies have also demonstrated that probiotic yeasts such as *Saccharomyces boulardii* remain viable during gastrointestinal transit and exert beneficial effects through antioxidant, anti-inflammatory, and immunomodulatory mechanisms (72, 79). Therefore, we evaluated the antioxidant potential of both crude acetone extract as well as the whole lyophilized cells of novel *R. paludigena* strain through DPPH and ABTS assays. The crude acetone extract of *Rhodotorula* exhibited strong antioxidant activity especially at concentrations above 0.025 μg/mL approaching a radical scavenging of more than 90%. However, antioxidant assay at similar extract concentration through ABTS assay revealed lower radical scavenging. This differential radical scavenging can be explained on the basis of solvent effects since DPPH is more soluble in acetone, the DPPH radical reacts efficiently with hydrophobic antioxidants. ABTS on the other hand exhibits slower reaction kinetic and reduced stability in organic solvents leading to underestimation of antioxidant activity (80, 81). In contrast to pigment extract, the whole lyophilized cells of *Rhodotorula* showed a higher radical scavenging in ABTS compared to DPPH reagent. This is because compared to DPPH, ABTS radical is water-soluble, smaller, and capable of interacting with cell-surface and membrane-associated antioxidants, resulting in comparatively higher ABTS activity. Similar observations have been reported for intact yeast and plant matrices, where DPPH underestimates antioxidant capacity unless compounds are fully extracted (81–83).

The *Rhodotorula* strain was isolated from a brackish water ecosystem; therefore, *Vibrio* spp. are one of the most prevalent fish pathogen in such ecosystems (84). Therefore, four of the five pathogens selected in the present study were those commonly associated with diseases in farmed finfish across different mariculture systems (85, 86). The crude acetone extract of *Rhodotorula* showed substantial antimicrobial and antibiofilm activity against all tested pathogens including *Vibrio harveyi, V. parahaemolyticus, V. alginolyticus, and Escherichia coli.* Carotenoids from *Rhodotorula glutinis* demonstrated strong antimicrobial and antibiofilm activity and induced a suppression of expression of quorum-sensing genes in against *Staphylococcus aureus* and *Salmonella typhimurium* (87). Keceli et al. (88) reported that eleven out of twenty different strains of *Rhodotorula glutinis* isolated from soil, plant and pine and tree leaves showed antibacterial effects against pathogenic bacteria like *S. aureus* and *E. coli*. Yeasts of the genus *Rhodotorula* have attracted attention for their potential probiotic properties, particularly due to their ability to produce bioactive carotenoids such as *β*-carotene, torulene, and torularhodin with strong antioxidant activity (47). These compounds may contribute to host health by modulating oxidative stress and microbial interactions within the gut. In addition, emerging evidence suggests that carotenoids can influence microbial adhesion and biofilm formation, which are critical determinants of intestinal colonization. For instance, carotenoid compounds have been shown to significantly reduce biofilm formation and alter cell surface hydrophobicity in lactic acid bacteria, thereby affecting their adhesion properties and colonization potential (89). Such findings indicate that carotenoid-producing yeasts like Rhodotorula may indirectly modulate gut microbial dynamics. However, as these effects are context- and strain-dependent, and given that some *Rhodotorula* species have been reported as opportunistic pathogens, their probiotic application warrants careful strain-level evaluation (49). Acetone extract of *R. mucilaginosa* exhibited an inhibition zone of about 10 mm against *S. aureus* (90). Carotenoids also act as antimicrobial agents and some reports have shown the potential of these molecules to be scaled up to industry (91) and some reports have attempted to employ metabolically engineered yeast to induce disease resistance against *Vibrio* spp. in shrimp (92). The antimicrobial ability of pigment-rich extracts can also translate to improved disease resistance of fish challenged with pathogens (86, 93). Therefore, our findings support the potential of *Rhodotorula* sp. for use as natural and safe antibiotic alternative in aquaculture.

The lipids and amino acids identified in the yeast biomass, can enhance probiotic efficacy through several mechanisms acting at the host-microbe and interface. Microbial-derived fatty acids are known to modulate gut epithelial integrity, reduce inflammation, and influence antioxidant signaling pathways (94, 95). These lipids, including short chain fatty acids that were identified in the fatty acid profile of the *Rhodotorula* strain also, can also act as metabolic substrates for commensal gut microbes, thereby indirectly supporting microbiome balance rather than functioning solely as host nutrients (96). Studies have also demonstrated that probiotic yeasts enriched in bioactive fatty acids improve gut microbiota profile in vertebrates (97). Although the lipid content of the *Rhodotorula* isolate was only about 2%, its fatty acid profile represented a remarkably high proportion of PUFAs, exceeding 25% of total fatty acids, with linoleic acid and *α*-linolenic acid dominated the PUFA profile. Dietary linoleic acid and α-linolenic acid are critical substrates for the action of elongase and desaturase enzymes that lead to the biosynthesis of EPA, DHA and ARA. EPA is often the limiting omega-3 fatty acid when replacing fish oil with microbial lipid sources such as thraustochytrids in aquafeeds (98). The presence of EPA in the *Rhodotorula* isolate indicates that the biosynthetic machinery is active in this isolate and can be employed in the future to increase the EPA production either through media optimization (99) or genetic modifications in the microbe as was achieved in other yeast species (100).

The amino acid profile of Rhodotorula sp. biomass shows both similarities and distinct differences when compared with conventional protein sources such as fish meal and soybean meal. Notably, *Rhodotorula* exhibited appreciable levels of key essential amino acids such as lysine and valine which are critical for fish growth and are often limiting in plant-based ingredients like soybean meal. Lysine constituted about 6.2% of the total dry matter in the isolated *Rhodotorula* strain. Lysine is essential for growth, feed efficiency, muscle protein synthesis, and general metabolic function in fish. This amino acid is often limited many plant-based alternative protein sources used in aquafeeds including soybean meal and corn gluten meal (101, 102). Although the overall amino acid concentrations were generally lower than fish meal, the profile of *Rhodotorula* was more balanced than soybean meal for certain essential amino acids. This suggests that *Rhodotorula* biomass could serve as a complementary protein source, potentially improving the amino acid balance when used in combination with plant-based feeds. The high crude protein and lysine content of *Rhodotorula* opens a possibility of using it as a supplementary feed additive along with the plant-based ingredients to offset the deficiency of lysine.

Though the study developed a reliable framework for *in vitro* evaluation of potential probiotics in aquaculture, there are limitations of the study that must be addressed in future studies. First of all, the chemical nature of the pigment has not been clarified. *Rhodotorula* spp. are known to simultaneously produce different types of pigments like *β*-carotene, Torulene, *γ*-carotene, and Torularhodin with their relative proportions varying with species and culture conditions (63, 64). Though a preliminary screening and temporal variation in pigment content was revealed in our study, it is possible that the crude acetone extract may have carried more than one species of pigment with distinct contribution towards bioactivity. An LC–MS based elucidation of the chemical composition of the crude *Rhodotorula* extract will enable us to decipher the novel compounds that are responsible for various bioactive effects observed in the study. This will further enable a better optimization of culture conditions towards the targeted production of the specific bioactive compounds. As far as the culture of *Rhodotorula* strain is concerned, the yield achieved in the present is much lower than is expected from an industrial scale microbe. Though we did not make any attempt to optimize the culture conditions of the strain for increased yield of pigment, lipid or EPA this will form an important area of focus if this strain is to gain any economic viability as aquaculture probiotic after *in vivo* testing.

## 5 Conclusion

We isolated and identified a novel strain of *Rhodotorula* sp. from mangrove ecosystems and assessed its probiotic potential through a series of *in vitro* investigations, including pigment production, antibacterial activity, antioxidant potential and viability in simulated gut environments. The isolate exhibited high pigment production and showed strong antibacterial activity against major fish pathogens of the genus *Vibrio*. The isolate also demonstrated strong antioxidant capacity and favorable nutritional profile characterized by high levels of PUFA (including EPA) and lysine. These findings indicate that the isolate has a strong probiotic potential in aquaculture applications. The polyphasic framework developed here can serve as a pre-screening strategy in future studies before *in vivo* trials on farmed aquatic species.

## Data Availability

The original contributions presented in the study are included in the article/Supplementary material, further inquiries can be directed to the corresponding author.
