# Supplementary material for: Polyphasic in vitro characterization of the pigment-producing microfungus Rhodotorula sp. for potential application as a probiotic in mariculture
Source: Front Nutr. 2026 May 7;13:1759183. doi: 10.3389/fnut.2026.1759183 (PMC13190513; doi:10.3389/fnut.2026.1759183)
Supplement: Supplementary file 1 [file Supplementary_file_1.DOCX]

**Polyphasic *in vitro* Characterization of the Pigment-Producing Microfungus *Rhodotorula* sp. for Potential Application as a Probiotic in Mariculture**

Qurat ul Ain^1^, Pooja Baiju^1^, Saima Rehman^1^, Kajal Chakraborty^1#^, Ashwin A Pai^1^, Chandrasekar S.^1^, Sanal Ebeneezar^1^, D. Linga Prabu^1^, Unnimaya T.D.^1,2^, Shylaja S.^1^, Adnan H. Gora^1*^

**Supplementary figures:**

**
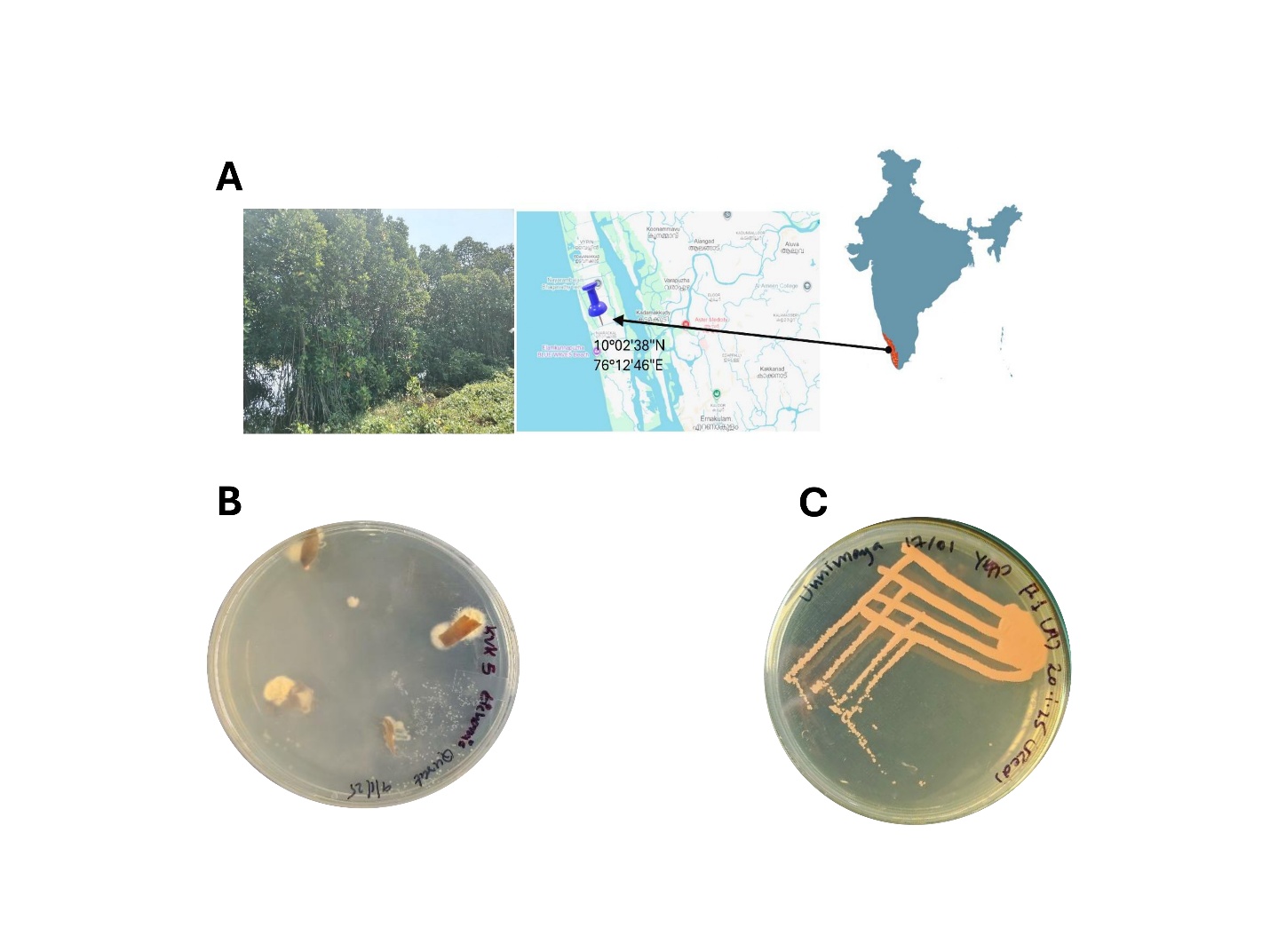
**

**Supplementary Figure 1:** Sample collection site and isolation of pigmented *Rhodotorula* *paludigena* strain. (A) Mangrove ecosystem at the sampling location along the southwest coast of India, with geographic coordinates (10°02′38″ N, 76°12′46″ E) indicated on the map; the inset highlights the sampling site within India. (B) Primary isolation plate showing heterogeneous microbial growth obtained from the environmental sample after incubation. Distinct colonies with varying morphology and pigmentation are visible. (C) Purified pigmented microbial isolate obtained after repeated streaking on agar medium, showing uniform colony morphology and characteristic orange pigmentation.

**
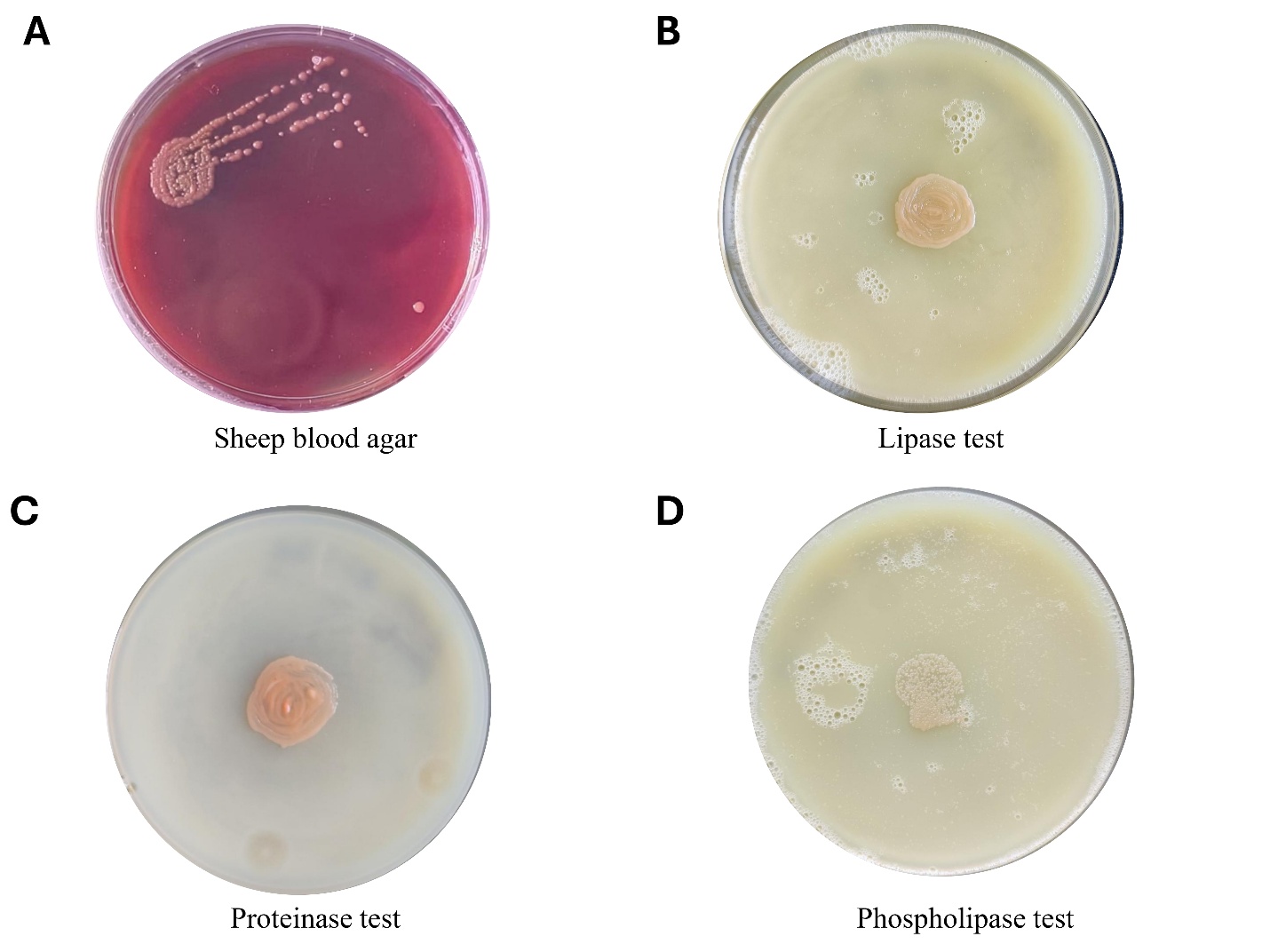
**

**Supplementary Figure 2: Hemolytic activity and extracellular enzyme production by the isolated *Rhodotorula paludigena* strain.**
(A) Growth of the isolate on sheep blood agar showing absence of a clear hemolytic zone around colonies, indicating non-hemolytic activity.
(B) Lipase activity assay showing no distinct halo or precipitation zone around the colony, suggesting a negative reaction under the tested conditions.
(C) Proteinase activity assay showing no visible clearance zone around the colony, indicating lack of detectable proteolytic activity.
(D) Phospholipase activity assay showing no evident precipitation or halo zone surrounding the colony, indicating a negative phospholipase reaction.

**
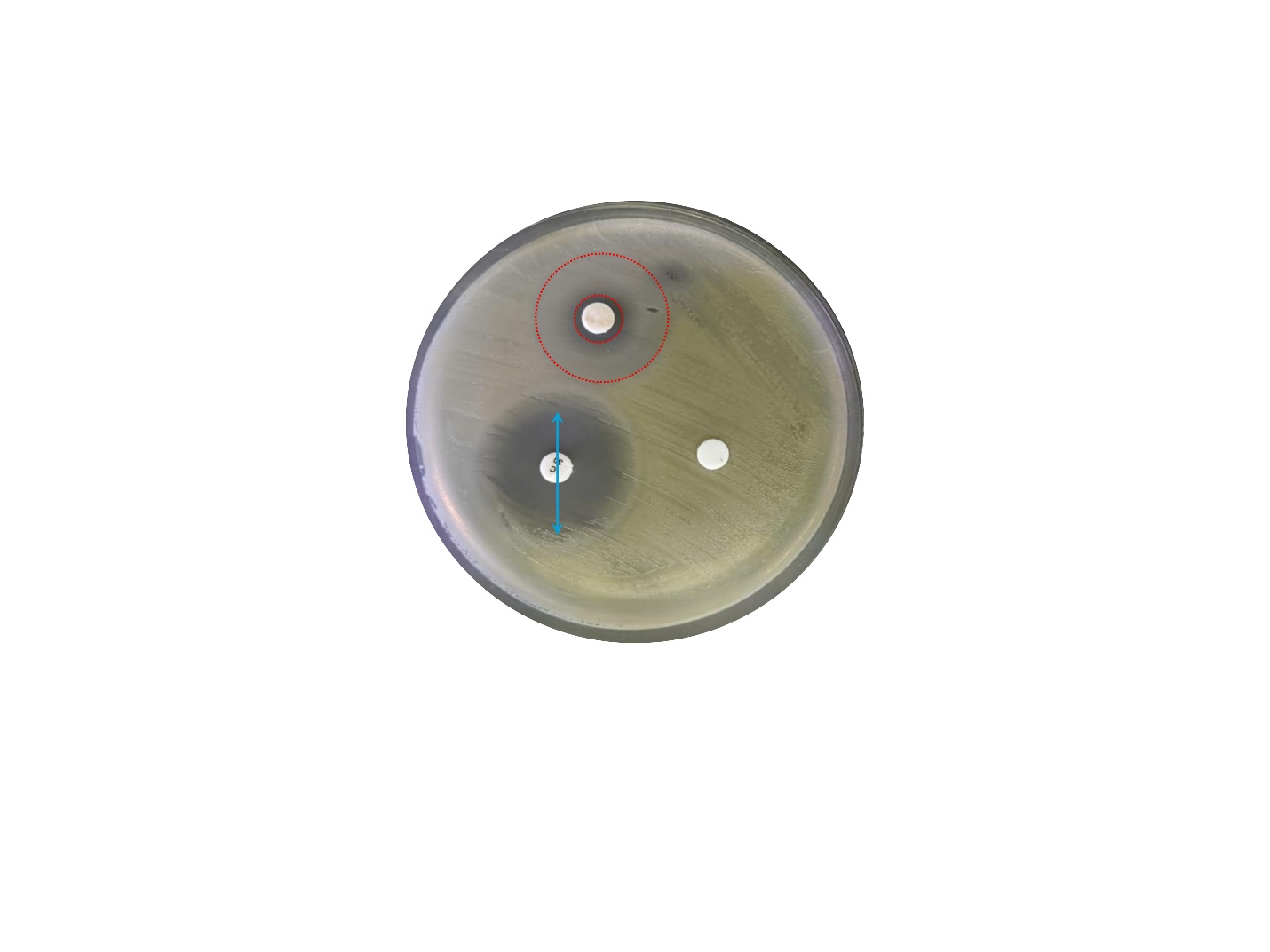
**

**Supplementary Figure 3:** Representative agar diffusion plates showing zones of inhibition (ZOI) produced by Rhodotorula sp. biomass or extract against a mixed culture of *E. coli, Vibrio parahaemolyticus, Vibrio alginolyticus, and Vibrio harveyi* (final OD_600_: 0.319± 0.003). The white discs represent controls, while red and blue arrows indicate the zones produced by *Rhodotorula* samples and antibiotic control, respectively.
